# Feeding the Skin Barrier: The Impact of Macro‐ and Micronutrients on Skin Barrier Function

**DOI:** 10.1002/clt2.70105

**Published:** 2025-11-18

**Authors:** Klaudia Ryczaj, Burcin Beken, Cezmi Akdis

**Affiliations:** ^1^ Children’s Clinical Hospital, University Clinical Centre, Medical University of Warsaw Warsaw Poland; ^2^ Department of Pediatric Allergy and Immunology Acibadem University School of Medicine Istanbul Türkiye; ^3^ Swiss Institute of Allergy and Asthma Research (SIAF) University of Zurich Davos Switzerland

**Keywords:** atopic dermatitis, diet, dietary patterns, nutrients, skin

## Abstract

**Background:**

Skin is the largest organ of the human body, and acts as a fundamental barrier. Beyond its protective role, it serves as a key immune organ, mediating immune surveillance and regulation. Exposure to environmental factors such as mechanical trauma, detergents, air pollution, and microbial dysbiosis can compromise the skin barrier triggering the release of pro‐inflammatory cytokines that contribute to the pathogenesis of allergic diseases including atopic dermatitis (AD). Nutrition profoundly impacts skin health, influencing cell proliferation, tissue repair, and immune functions.

**Methods:**

This review aims to explore the relationship between diet and skin barrier function, with a specific focus on AD.

**Results:**

The evidence on micro‐ and macronutrients, probiotics, and various dietary patterns, highlighting their potential to enhance or impair skin barrier integrity, provides a comprehensive exploration of how diet may serve as a modifiable factor in supporting skin health and preventing allergic diseases. This review also outlines directions for improving future research.

**Conclusion:**

Diet is an important modifiable factor in preserving skin barrier integrity and may contribute to the prevention and management of AD. However, inconsistent evidence precludes definitive dietary recommendations, highlighting the need for further research.

## Graphical abstract

Created in BioRender. Ryczaj, K. (2025) https://BioRender.com/svad2e8.

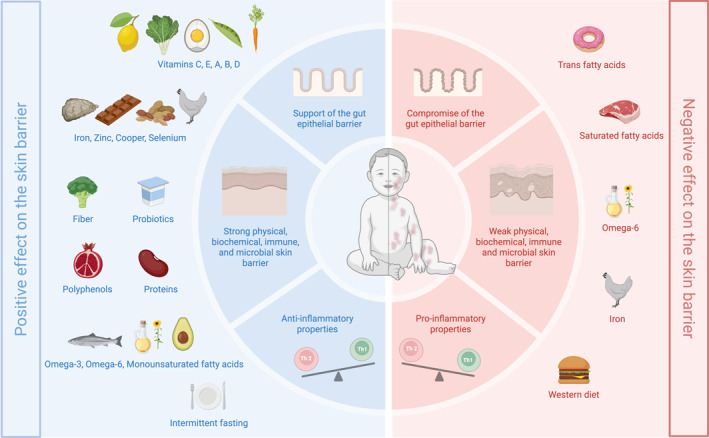

## 1 Introduction

Skin, the largest and outermost organ of the human body, acts as a fundamental mechanical and immunological barrier, protecting against pathogens, allergens, environmental toxins, pollutants, and irritants. Beyond its protective role, skin serves as a key immune organ, mediating immune surveillance and regulation. Maintaining the integrity of its barrier function is essential for health; however, exposure to environmental factors such as mechanical trauma, detergents, air pollution, and microbial dysbiosis can compromise the skin barrier, triggering the release of pro‐inflammatory cytokines such as IL‐1, IL‐25, IL‐33, TSLP and multiple chemokines. These cytokines activate immune cells, including dendritic cells and T cells, driving Th2‐dominant immune responses and contributing to the pathogenesis of allergic diseases [1].

Atopic dermatitis (AD), a chronic skin disease characterized by persistent barrier dysfunction and inflammation, exemplifies the consequences of impaired skin integrity. AD is often the first step of the “allergic march,” which serves as a risk factor for developing food allergies (FA), asthma, and allergic rhinitis. Researches highlight that early‐life exposure to food allergens through damaged skin, rather than oral exposure via a healthy gastrointestinal tract, can result in epicutaneous sensitization and subsequent IgE‐mediated FA [2]. Even subclinical barrier damage, such as increased transepidermal water loss (TEWL), has been independently associated with food sensitization, emphasizing the critical role of a healthy skin barrier in preventing allergic diseases [3].

The recently proposed The Epithelial Barrier Theory further underscores the skin's importance in health and diseases, attributing rising rates of allergic, autoimmune, and chronic inflammatory conditions that result in a damage across epithelial barriers in the skin, respiratory tract, and gastrointestinal tract [4]. Strategies to strengthen the skin barrier, such as emollients, microbiome modulation, and dietary interventions, are gaining attention as promising preventive approaches.

Nutrition profoundly impacts skin health, influencing cell proliferation, tissue repair, and immune functions. Macro‐ and micronutrients derived from food contribute to skin barrier integrity and immune modulation [5, 6]. Conversely, poor dietary quality or malnutrition can exacerbate skin damage and immune dysregulation [7, 8, 9]. Evidence linking nutrient deficiencies to skin disorders further emphasizes the need to understand how dietary components affect skin health [10, 11, 12, 13].

This review aims to explore the intricate relationship between diet and skin barrier function, with a specific focus on AD. By synthesizing evidence on dietary micro‐ and macronutrients, other compounds derived from whole foods, dietary patterns, and skin barrier‐damaging substances present in the diet, this review will highlight the potential of nutrition to enhance skin barrier integrity, prevent allergic diseases, and complement existing therapeutic approaches. This review will provide a comprehensive analysis of how diet can serve as a modifiable factor in promoting skin health and preventing allergic conditions. Detailed information on micro‐ and macronutrients and diet patterns affecting the skin barrier is presented in the Supporting Information S1, while their associations with AD are discussed in the main text below.

## 2 Nutrients

### 2.1 Micronutrients

#### 2.1.1 Vitamins

##### 2.1.1.1 Vitamin C

Vitamin C, also known as L‐ascorbic acid, is a water‐soluble vitamin that plays a crucial role in maintaining skin health. Since humans cannot synthesize vitamin C endogenously, obtaining sufficient amounts through diet is essential. Rich dietary sources include fresh fruits and vegetables such as citrus fruits (grapefruits, oranges, lemons), cherries, kiwifruit, spinach, broccoli, and red peppers. Due to its sensitivity to heat, the vitamin's nutritional value decreases with cooking or boiling, making raw sources particularly important for maintaining optimal intake.

**Clinical relevance in AD**

*AD prevention*

Oxidative stress is also increasingly recognized as a significant factor in the pathophysiology of allergic conditions, including AD. [14]. Systematic review of observational studies suggest a positive correlation between antioxidant levels and the prevalence of eczema, underscoring the potential protective role of antioxidants in these conditions [15]. Moreover, an observational study found that higher concentrations of vitamin C in breast milk have been associated with a reduced risk of AD in infants, suggesting that adequate maternal vitamin C intake may confer protective benefits against the condition [16].

*AD treatment/management*

Specific to vitamin C, observational research has identified reduced plasma levels of the vitamin in AD patients, accompanied by lower epidermal ceramide levels, pointing to its role in maintaining skin barrier function [17].

##### 2.1.1.2 Vitamin E

Vitamin E refers to a group of eight fat‐soluble compounds, with α‐tocopherol and γ‐tocopherol being the most biologically significant. α‐Tocopherol is predominantly sourced from green leafy vegetables and oils such as olive and sunflower oil, while γ‐tocopherol is abundant in canola and soybean oils [14]. Notably, these two forms exhibit distinct biological effects: α‐tocopherol is renowned for its anti‐inflammatory properties, whereas γ‐tocopherol has been observed to promote inflammatory responses [14].

**Clinical relevance in AD**

*AD treatment/management*

In the context of AD, evidence suggests that vitamin E supplementation can improve symptoms and enhance the quality of life for patients, without significant side effects. A randomized controlled trial (RCT) proposed a daily dose of 400 IU as a safe and effective intervention for managing AD symptoms [18]. Moreover, a single‐blind clinical study indicate that vitamin E supplementation at a dose of 400 IU (268 mg) may reduce total IgE levels in patients with AD, further supporting its therapeutic potential in mitigating allergic inflammation [19]. However, a cross‐sectional study of children aged 0–24 months diagnosed with AD found no association between dietary vitamin E intake and serum IgE levels; instead, serum vitamin E levels showed a significant inverse relationship with serum IgE levels [20].

The intake of vitamin E in these studies was higher than the values of the dietary reference intakes. According to EFSA, the adequate intake (AI) of vitamin E is 5–11 mg/day for children and 11 mg/day (approximately 16.5 IU) for adults [21].

##### 2.1.1.3 Vitamin A

Vitamin A is a fat‐soluble vitamin essential for maintaining skin health and immune functions. It is derived from two primary dietary sources: retinoids from animal‐based foods such as fish, liver, dairy products, and eggs, and provitamin A carotenoids from plant‐based foods like carrots, sweet potatoes, and orange or yellow vegetables. After ingestion, provitamin A is converted into active retinoids. Cooking methods such as grinding, steaming, boiling, and stir‐frying with oil (but not deep‐frying) enhance carotenoid absorption, and combining vitamin A‐rich foods with protein‐rich meals further improves bioavailability [22].

**Clinical relevance in AD**

*AD treatment/management*

In patients with AD, studies have reported lower levels of vitamin A and its derivatives [23]. Co‐deficiency of vitamin A and vitamin D has been associated with more severe AD presentations in children, as demonstrated in an observational clinical study [24]. Additionally, vitamin A deficiency increases the risk of *Staphylococcus aureus* infections [25], a common trigger of AD flares.

##### 2.1.1.4 Vitamin B

The B vitamins, a group of water‐soluble compounds, play diverse roles in maintaining skin integrity and function. This group includes thiamine (B1), riboflavin (B2), niacin (B3), pyridoxine (B6), biotin (B7), folic acid (B9), and cobalamin (B12). Rich dietary sources include eggs, meat, fish, legumes and fortified foods, which have made deficiencies relatively uncommon in developed countries [23]. However, when deficiencies do occur, they can compromise skin health and function.

**Clinical relevance in AD**

*AD prevention*

Conversely, dietary intake of niacin has not been shown to confer protective effects against AD, based on finding from a prospective cohort study [26].

*AD treatment/management*

A deficiency in vitamin B12 has been linked to increased severity of AD [27, 28], with a single case report documenting significant improvement in a patient with severe refractory AD following B12 supplementation [28].

##### 2.1.1.5 Vitamin D

Vitamin D is primarily synthesized in the skin, where UVB radiation converts 7‐dehydrocholesterol into cholecalciferol (vitamin D3). This precursor undergoes hydroxylation in the liver and kidneys to produce the active hormone, 1,25‐dihydroxyvitamin D3 (calcitriol). Although small amounts of vitamin D are obtained from dietary sources such as fatty fish, egg yolks, and fortified foods, approximately 80% of the body's vitamin D requirements are met through sun exposure [22, 29].

**Clinical relevance in AD**

*AD prevention*

The genetic landscape significantly influences vitamin D's role in skin health. Polymorphisms in the Vitamin D Receptor (VDR) gene and mutations in the FLG gene have been implicated in AD susceptibility [30]. Vitamin D deficiency has been linked to an elevated risk of AD, particularly in obese individuals, based on findings from an observational study [31].

*AD treatment/management*

A meta‐analysis of observational studies and RCTs found lower serum vitamin D levels in AD patients [32], with evidence from a observational study suggesting an association between reduced 25‐hydroxyvitamin D levels and increased AD severity [33]. However, several other observational studies have not corroborated these findings [34, 35]. Vitamin D supplementation has shown promise in alleviating AD symptoms. Systematic reviews and meta‐analyses indicate that supplementation is generally safe, well‐tolerated, and effective in reducing AD severity [32, 36, 37, 38]. Daily doses of approximately 1600 IU of vitamin D have been reported to significantly improve disease outcomes [36]. Notably, in a RCT, direct sun exposure has been found to be even more effective than supplementation (400 IU/d) in reducing the incidence of infant eczema [39]. According to EFSA, the adequate intake (AI) of vitamin D is 15 μg/day (600 IU/day) for children and adults and 10 μg/day (400 IU) for infants [21].

#### 2.1.2 Minerals

##### 2.1.2.1 Iron

Iron is an essential micronutrient with important roles in maintaining skin integrity, immune function, and overall health. Dietary sources of iron include red meat, poultry, fish, legumes, and fortified cereals. Heme iron, primarily derived from animal‐based foods, is more efficiently absorbed than non‐heme iron, which is predominant in plant‐based diets. The bioavailability of dietary iron can be influenced by various factors. For instance, high calcium intake may moderately inhibit iron absorption, while vitamin C enhances non‐heme iron bioavailability by up to threefold. Foods rich in phytates, such as lentils, can significantly impair the absorption of non‐heme iron. However, incorporating oily fish and vitamin C into a phytate‐rich diet has been shown to counteract these inhibitory effects, improving iron uptake [23].

**Clinical relevance in AD**

*AD prevention*

Prenatal iron status appears particularly influential, with maternal iron and folic acid supplementation during pregnancy reducing AD risk by up to 80% by the age of six, according to an observational study [40].

*AD treatment/management*

Observational studies suggest an association between iron deficiency and AD. [41, 42].

##### 2.1.2.2 Zinc

Zinc is an essential trace mineral predominantly obtained from animal‐based foods such as meat, poultry, fish and oysters. Similar to iron, zinc deficiency often co‐occurs with iron deficiency [23].

**Clinical relevance in AD**

*AD prevention*

Dietary zinc intake also shows an inverse relationship with the risk of childhood eczema, highlighting the importance of adequate zinc levels in mitigating AD risk, based on an observational study [43].

*AD treatment/management*

According to a review by Ogawa et al. zinc deficiency in AD patients has been linked to elevated skin pH, likely due to reduced UCA production [44]. A meta‐analysis of observational studies and RCTs confirmed that individuals with AD exhibit lower zinc levels in serum, hair, and erythrocytes [45]. Furthermore, several observational studies suggest a correlation between serum zinc concentrations and AD severity, with lower zinc levels associated with more advanced disease [46].

##### 2.1.2.3 Copper

Copper is sourced from a variety of dietary staples, including beef liver, oysters, crabs, mushrooms, dark chocolate, fish, turkey and chicken meat, as well as nuts.

**Clinical relevance in AD**

*AD treatment/management*

An observational study reports low copper levels in pediatric skin diseases [47]. However, another observational studies by Toyran et al. indicate no significant differences in serum copper concentrations between healthy children and those with AD. [48].

### 2.2 Macronutrients

#### 2.2.1 Carbohydrates

##### 2.2.1.1 Fiber

Dietary fiber, a key component of many plant‐based foods, is crucial for maintaining a balanced gut microbiome. Prebiotics, a subset of dietary fibers, are indigestible food components that selectively stimulate the growth and activity of beneficial gut bacteria. While not all fibers qualify as prebiotics, fermentable fibers such as inulin, fructo‐oligosaccharides (FOS), galacto‐oligosaccharides (GOS), and xylooligosaccharides (XOS) have been extensively studied for their immune‐modulating properties [49, 50]. These fibers are abundant in foods like garlic, onions, asparagus, broccoli and whole grains. Prebiotics serve as a substrate for the gut microbiota, enabling the production of short‐chain fatty acids (SCFAs) during fermentation.

**Clinical relevance in AD**

*AD prevention*

Another systematic review suggested that early‐life exposure to SCFAs might provide a protective effect against the development of AD [51].

*AD treatment/management*

Several cross‐sectional studies and animal research have shown the potential benefits of fiber‐rich diet in managing atopic dermatitis and allergic diseases [52, 53, 54]. One systematic review identified a study that investigated the effects of FOS in infants with AD, reporting a significant improvement in SCORAD scores compared to a placebo group [37].

#### 2.2.2 Fats

Fats are crucial for maintaining skin barrier function, particularly through their role in the lipid matrix of the stratum corneum, which comprises ceramides (50%), cholesterol (25%), and free fatty acids (15%). Disruptions in lipid balance, whether due to dietary imbalances or deficiencies, can compromise skin barrier integrity, increase TEWL, and amplify susceptibility to inflammation [11, 55].

Attributing the effects of specific fatty acids is challenging, as oils typically contain a complex mix of various fatty acid species. This natural co‐occurrence of multiple fatty acids within oils complicates isolating the unique contributions of each to skin health.

Moreover, recent studies suggest that intestinal microbes may further influence fatty acid availability and structure. In a mouse model, gut microbes such as *Lactobacillus plantarum,* a nonpathogenic bacterium commonly found on fresh fruits and vegetables, were shown to metabolize polyunsaturated fatty acids (PUFAs) into various intermediates, which may influence the host's fatty acid profile [56].

##### 2.2.2.1 Polyunsaturated Fatty Acids (PUFAs)

PUFAs, particularly essential fatty acids (EFAs) such as omega‐3 and omega‐6, are vital for skin health. As EFAs cannot be synthesized by the body, they must be obtained through diet.

Omega‐3 fatty acids, including alpha‐linolenic acid (ALA), eicosapentaenoic acid (EPA), and docosahexaenoic acid (DHA), offer extensive benefits for the skin. ALA is derived from plant‐based foods like flaxseeds, walnuts, and chia seeds, while EPA and DHA are primarily sourced from oily fish such as salmon, mackerel, and sardines or through fish oil supplements.

Omega‐6 fatty acids, particularly linoleic acid (LA) and γ‐linolenic acid (GLA), are essential for maintaining skin health. LA is predominantly found in vegetable oils such as corn, sunflower, and soybean oils, while GLA is derived primarily from evening primrose oil (which contains approximately 70% LA and 10% GLA), borage seed oil, and hemp seed oil.

In an optimal skin barrier, the omega‐6 to omega‐3 ratio is approximately 3:1 [55]. The synthesis of omega‐3 and omega‐6 derivatives relies on the same enzymes, leading to a phenomenon known as *enzyme competition*. Due to the high prevalence of omega‐6 fatty acids in modern diets, omega‐3 derivatives may be underrepresented in skin lipid profiles, as omega‐6 metabolites tend to dominate enzymatic pathways [57].

Interactions between genetics and nutrition can affect fatty acid metabolism and the risk of atopic diseases like atopic dermatitis. Variants in the *FADS* gene cluster influence long‐chain PUFA levels and eczema risk, with dietary responses potentially depending on genetic profiles. For example, gestational fish intake affects DNA methylation and expression of *FADS1/2* and *ELOVL5* genes, linking maternal LCPUFA consumption to reduced allergy risk in offspring. These findings highlight the need to include genetic factors in future dietary intervention studies [58, 59].

**Clinical relevance in AD**

*AD prevention*

Based on systematic review of RCTs, maternal omega‐3 supplementation during pregnancy may provide benefits for children under 3 years, potentially reducing eczema incidence in this subgroup, particularly among children at high risk of atopy [60, 61]. In the included studies, the amount of daily ω‐3 PUFAs supplemented ranged from 400 to 4500 mg [60, 61]. While an observational study has associated increased fish intake during infancy with a reduced risk of AD [62], a systematic review of RCTs and prospective cohort studies has found that supplementation of omega‐3 during childhood have not consistently demonstrated a preventive effect against AD [63]. The daily doses used in the included studies ranged from 184 to 650 mg of DHA, or formulas containing 17 mg of DHA per 100 kcal. Similarly, a meta‐analysis of double‐blinded RCTs reported no efficacy of omega‐6 supplementation in preventing the onset of AD [64]. In the included studies, GLA was administered at doses ranging from 40 to 100 mg per day.

The interaction between genetics and nutrition is further illustrated by findings that gestational fish intake can affect DNA methylation and the expression of *FADS1/2* and *ELOVL5* genes, which are associated with allergic outcomes in children. These results suggest a mechanistic link between maternal intake of long‐chain PUFAs and reduced allergy risk in offspring [59].

*AD treatment/management*

A systematic review of RCTs included one study using DHA at a daily dose of 5.4 g and found that the therapeutic use of omega‐3 fatty acids for managing AD has not shown improvements in AD severity, while two studies on fish oil suggested a possible modest benefit [65]. Likewise, a Cochrane analysis found no significant benefits from omega‐6‐rich dietary supplements, such as sunflower or hemp seed oils [65], and insufficient evidence to support oral supplementation with evening primrose oil and borage oil [66].

According to EFSA, the adequate intake (AI) of DHA is 250 mg/day for children and adults, 100 mg/day for infants, and an additional 100–200 mg/day of preformed DHA is recommended for pregnant and lactating women [21].

According to the EAACI position paper, supplementation with omega‐3 could be recommended for preventing and treating AD, with some studies showing beneficial effects and no reported adverse effects [58].

##### 2.2.2.2 Saturated Fats (SFAs)

SFAs, predominantly found in fatty meats, butter, eggs, full‐fat dairy products, palm oil, and coconut oil, have been implicated in oxidative stress and inflammation.

**Clinical relevance in AD**

*AD prevention*

High meat consumption has also be linked to increased AD prevalence [67]. Notably, early exposure to SFAs during infancy has been identified as a potential risk factor for the development of AD, suggesting that dietary patterns early in life may influence long‐term skin health and immune function, according to a review by Kong et all [68].

*AD treatment/management*

The relationship between SFAs and AD has been further explored in an observational clinical study, revealing a strong association between high meat consumption, one of the richest sources of SFAs, and increased AD severity [69]. Additionally, exacerbation of AD has been linked to early exposure to SFAs during infancy [68].

##### 2.2.2.3 Trans Fatty Acids (TFAs)

TFAs, predominantly found in hydrogenated vegetable oils used in margarine, processed snacks, deep‐fried foods, and baked goods, have been strongly implicated in pro‐inflammatory effects and immune dysfunction.

**Clinical relevance in AD**

*AD prevention*

The dietary prevalence of TFAs, particularly in processed foods such as fast food and margarine, has been associated with a heightened risk of AD in children and adolescents, based on a birth cohort study and a review of cross‐sectional and case control studies [70, 71].

*AD treatment/management*

Notably, recent research based on a cross‐sectional study underscores a direct connection between high dietary TFAs consumption and the exacerbation of AD symptoms [69], highlighting the detrimental impact of these fats on skin health.

### 2.3 Other Compounds

#### 2.3.1 Probiotics

Probiotics are non‐pathogenic microorganisms which exhibit beneficial effects when given in adequate numbers. They are found in foods such as yogurt, kimchi, cheeses, pickles, kombucha, and sauerkraut.

**Clinical relevance in AD**

*AD prevention*

A meta‐analysis investigating curative effects of probiotics, 6 clinical trials with 1581 participants were analyses for preventive effects. *Lactobacillus rhamnosus GG* was the used probiotic strain in all 6 studies and five studies found significant reduction in AD incidence at 2 years of age in intervention groups [72]. Meta‐analyses of these 6 preventions clinical trials indicated probiotics could be effective for prevention. They found a risk reduction of 61% associated with the use of prenatal and/or postnatal probiotics for AD [72]. Although there are some clinical evidences that prenatal use of probiotics might be effective in preventing AD; the studies are not enough to recommend probiotics for preventing due to the heterogeneity of these studies in terms of the studied species, dosage, and the treatment protocol.

*AD treatment/management*

The efficacy of probiotics in treating AD has shown mixed results in clinical studies. A Cochrane review conducted in 2008 concluded that probiotics are not effective in treating AD and probiotic treatment carries a small risk of adverse events such as infections and bowel ischemia [73]. The same group revised their analysis in 2018 and reported that currently available probiotic strains (*Lactobacillus* and *Bifidobacteria* species) probably make little or no difference in improving eczema symptoms [74]. A recent EAACI Task Force report (a meta‐analysis of 20 RCTs) probiotics (mostly *Lactobacillus species*) alone or combination with prebiotics or postbiotics showed a significant reduction in SCORAD scores, suggesting a reduction in AD symptoms in children without food allergies [37]. However, current evidence is insufficient to support the use of probiotics for treating AD, mainly due to variations in the bacterial strains studied, dosages used, and treatment protocols across the available studies.

#### 2.3.2 Polyphenols

Polyphenols are naturally occurring compounds found in foods such as green tea, cocoa, pomegranates, berries, apples, broccoli, onions, and coffee. Their levels in foods can be significantly reduced by cooking methods such as boiling, microwaving, and frying. The gut microbiota also plays an essential role in the absorption and metabolism of polyphenols [10]. Based on their chemical structures, polyphenols are primarily categorized into four groups: phenolic acids, flavonoids, stilbenes, and lignans. Among these, flavonoids are subdivided into flavones, flavonols, isoflavones, and flavanones. Commonly recognized polyphenols include resveratrol, quercetin, curcumin, epigallocatechin gallate, catechin, hesperetin, cyanidin, procyanidin, caffeic acid, and genistein.

**Clinical relevance in AD**

*AD treatment/management*

Clinical studies highlight the potential therapeutic benefits of polyphenols for AD. For instance, (193) pilot interventional clinical study on apple polyphenols indicated a reduction in AD‐associated symptoms, including inflammation, itching, and sleep disruption, along with lower eosinophil counts in peripheral blood [75].

## 3 Diet Patterns/Types

### 3.1 Western Diet

The Western diet, characterized by high consumption of processed and refined foods, saturated fats, trans fats, glycemic foods, and low intake of antioxidant‐rich fruits and vegetables making it energy‐dense but nutrient‐poor [14]. Moreover, it is associated with an excessive omega‐6 to omega‐3 fatty acid ratio, often exceeding 10:1, while food processing and cooking methods frequently lead to the loss or oxidation of omega‐3 fatty acids [76].

— Processed foods are foods that have been altered from their natural state, typically to increase their shelf life or improve their taste. Examples include fast foods, packaged snacks, and sugary drinks.
— Processed foods and specific cooking methods, such as grilling, frying, and baking, are significant contributors to the formation and accumulation of advanced glycation end products (AGEs), which play a role in skin inflammation. AGEs are complex molecules formed through the Maillard reaction, a non‐enzymatic interaction between reducing sugars and proteins or lipids, occurring naturally in the body but exacerbated by high‐temperature cooking and food processing [50].
— Refined foods are those that have undergone processing to remove their inherent fiber and nutrients, examples of which include white flour, white rice, and added sugars [77].

**Clinical relevance in AD**

*AD prevention*

Emerging evidence suggests that exposure to ultra‐processed foods correlates with an increased risk of allergic diseases, including AD. [50, 78, 79].

*AD treatment/management*

Notably, a substantial proportion of infants with severe AD exhibit sensitivity to tartrazine, a synthetic food dye commonly present in ultra‐processed products [80].

### 3.2 Mediteranian Diet

The Mediterranean diet, a traditional dietary pattern common in non‐Western countries, is characterized by a high intake of fruits, vegetables, whole grains, legumes, olive oil, and nuts. This diet is renowned for its health benefits and is associated with enhanced immune functions, owing to its abundance in macro‐ and micronutrients. Its high content of antioxidants, dietary fibers, and healthy fat sources, such as olive oil and fish oil, are thought to contribute to its anti‐inflammatory and antioxidant effects [37].

**Clinical relevance in AD**

*AD prevention*

The Mediterranean diet is considered protective against allergic diseases such as AD [37]. However, research findings on the relationship between adherence to the Mediterranean diet and allergic conditions like AD are inconsistent. For instance, while some studies suggest a protective effect, Castro‐Rodriguez et al. concluded in their review that the Mediterranean diet does not have a significant effect on preventing atopic eczema or atopy [81].

### 3.3 Plant‐Based Diet

Plant‐based diets, including vegan and vegetarian regimens, are recognized for their anti‐inflammatory and antioxidant properties, which can positively influence skin health.

— The vegetarian diet encompasses various dietary patterns that exclude meat, poultry, and fish but may include animal‐derived products such as dairy, eggs, and honey, depending on the specific subtype.
— The vegan diet, the most restrictive form of vegetarianism, excludes all foods derived wholly or partly from animals.

However, the impact of these diets on skin health varies depending on their composition and nutritional balance.

**Clinical relevance in AD**

*AD prevention*

The introduction of plant‐based foods during weaning was associated with favorable microbiota development. Higher levels of *Bifidobacteria* at 6 months and greater relative abundance of butyrate‐producing bacteria at 12 months were linked to a lower risk of AD [82].

*AD treatment/management*

Evidence from an observational study suggests that vegetarian diets can reduce AD symptoms, decreasing peripheral eosinophil counts and inhibiting prostaglandin E2 (PGE2) synthesis [83].

## 4 Maternal Diet

Maternal diet could be a target for improving preventive strategies for allergic diseases including AD which is the first step of atopic march. We know that there is a strong association between diet and gut microbiome [84, 85]. Moreover, allergic diseases and maternal diet may affect the child's microbiome directly [86] or indirectly via the maternal microbiome [87]. However, it's not easy to perform studies investigating the association between maternal diet during pregnancy and offspring allergy outcomes due to agnostic reasons. Although there is not a human study investigating the direct effect of maternal diet on epithelial barrier, we can have an indirect knowledge from studies investigating the association between maternal diet and allergy outcomes in the offspring.

Vegetarian diet has been very popular in recent years due to reduced risk of several diseases including obesity, diabetes, hypertension, heart diseases, and cancers. Vegetarian diet during pregnancy has also positive effects on allergy outcomes. In a recent study from Taiwan, the relationship between maternal vegetarian diets during pregnancy and the offspring occurrence of AD was investigated in a large study population including 20,172 mother‐child pairs. When 408 mothers who followed a vegetarian diet during their pregnancy were compared to 4080 non‐vegetarian mothers, the vegetarians showed a lower risk of developing AD before 18 months of age (OR = 0.65, 95% CI = 0.45–0.93, *p* = 0.018) [88].

Higher maternal vegetable and yogurt intake has been found to be related to decreased allergy outcomes such as AD, allergic rhinitis, asthma but not food allergy in the offspring at 4 years of age [89]. Higher vegetable and yogurt intake has also been reported to be associated with increased gut microbiome diversity and higher butyrate levels which influence immune development in utero via epigenetic modifications [90, 91]. There are also several studies that found an association between higher fruit and vegetable intake and decreased risk of atopic diseases including AD. [89, 92, 93, 94]. This could be due to increased microbiome diversity in these mothers [95] or the antioxidant properties of vegetables and fruits that may play a role against allergic diseases in the offspring [96]. (Table 1).

**TABLE 1:** Maternal diet and allergic diseases.

	Maternal dietary patterns	
	Reduced risk	Increased risk
Atopic dermatitis	Yogurt [89] Probiotic milk products [97]	Fast food [98]
	Fruits and vegetables [89, 92, 94]	Red meat [99, 100]
	Fİsh [98]	Shellfish [98]
	Supplementation of vitamin E, calcium, zinc, copper, magnesium and beta‐carotene [98]	
Allergic sensitization	Supplementation of vitamin C, vitamin D, copper [98]	Free sugar [98, 101]
	Fish [98]	Fatty fish [98]
	Mediterrenean diet [98]	Vegetable fat [98]
	Noodle diet [98]	Margarine [98]
	Omega‐3 fatty acid [98, 102]	Fruit, celery, sweet pepper [98]

Furthermore, consuming more meat during pregnancy was found to be associated with increased risk of atopic diseases in childhood [99, 100]. This may be attributed to the carcinogenic component that are generated during the preparation of meat at high temperatures [103] or intake of fatty acids which are related to allergic inflammation [104].

Studies investigating the dietary patterns during pregnancy found no association between dietary pattern including the Alternative Healthy Eating Index for Pregnancy (AHEI‐P), HEI [105, 106, 107] Mediterranean Diet Index (MedDI) [93, 107, 108] or intake of AGEs [109] and AD. However, maternal diet index (including more vegetables and yogurt, less rice & grains fried potatoes, red meat, %100 fruit juice, and cold cereals) (MDI) and infant diet diversity (IDD) seem more important in preventing AD [89, 98, 110, 111]. Both MDI and IDD have individual and joint associations with reduced risk of AD. Moreover, a diverse diet is correlated with greater microbial diversity in the human gut, which may contribute to immune regulation [112].

Maternal diet during pregnancy appears to play a significant preventive role, independent of the child's genetic predisposition. Studies have shown that children with FLG mutations had a similar risk of developing AD as those without the mutation whose mothers did not adhere to an allergy‐preventive diet. Importantly, the presence of the FLG mutation did not diminish the protective effect of maternal nutrition, highlighting that a balanced, anti‐inflammatory diet during pregnancy may mitigate the risk of AD regardless of genetic background [89].

## 5 Discussion

### 5.1 What Do We Know?

#### 5.1.1 Prevention of AD

There is growing evidence that early‐life nutrition, particularly during pregnancy and infancy, plays a pivotal role in the development of allergic diseases, including AD. Higher maternal intake of vitamin C, D, zinc, iron, fruits, vegetables, and fermented dairy (e.g., yogurt), as well as adherence to vegetarian or Mediterranean dietary patterns, has been associated with a reduced risk of AD in offspring. Maternal diet may shape the infant's immune system both directly and via modulation of the maternal and neonatal microbiome. Likewise, early‐life exposure to dietary fiber, prebiotics, and omega‐3 fatty acids may promote immune tolerance and lower the risk of allergic sensitization. In contrast, diets rich in UPFs, saturated fats, and added sugars may increase the risk of AD by contributing to gut barrier dysfunction, low‐grade inflammation, and nutrient deficiencies. Notably, UPFs often lack beneficial compounds and are subjected to high‐heat processing that degrades essential nutrients. Although systematic reviews indicate that specific nutrients alone may not consistently prevent allergic diseases, overall dietary quality and diversity appear to be important preventive factors.

#### 5.1.2 Management of AD

Several nutrients and dietary patterns show promise in alleviating AD symptoms. Supplementation with vitamin D, E, B12, zinc, and iron has been associated with improvement in disease severity, enhancement of skin barrier function, and modulation of inflammatory responses. Fiber and polyphenol‐rich foods may help reduce pruritus and skin inflammation. Plant‐based and Mediterranean diets can support symptom control by promoting microbiome diversity and immune regulation. On the other hand, high intake of saturated and trans fats, added sugars, and UPFs may exacerbate AD by promoting systemic inflammation and impairing barrier function.

## 6 Conclusion

**FIGURE 1:**
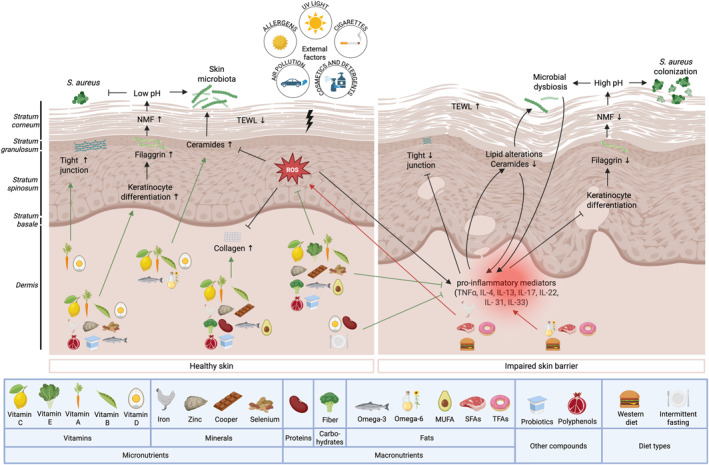
The relationship between nutrients, dietary patterns and the skin barrier. This figure provides a graphical overview of how dietary components influence the physical, biochemical, and immunological aspects of the skin barrier. The disruption of the epidermal barrier, triggered by external stressors and oxidative stress, initiates the release of pro‐inflammatory mediators. Such immune dysregulation contributes to impaired keratinocyte differentiation, alterations in lipid composition, a reduction in filaggrin protein levels, and increased TEWL. These changes create a conducive environment for *Staphylococcus aureus* colonization and cutaneous dysbiosis, ultimately compromising skin barrier function. Essential nutrients, including vitamins, minerals, fiber, proteins, polyunsaturated fatty acids (PUFAs), and monounsaturated fatty acids (MUFAs), probiotics, polyphenols and intermittent fasting play a supportive role in maintaining skin health. Conversely, excessive intake of omega‐6 fatty acids, saturated fatty acids (SFAs), trans fatty acids (TFAs), and Western diet are associated with skin barrier disruption (Created in BioRender. Ryczaj, K. (2025) https://BioRender.com/j05b417).

**FIGURE 2:**
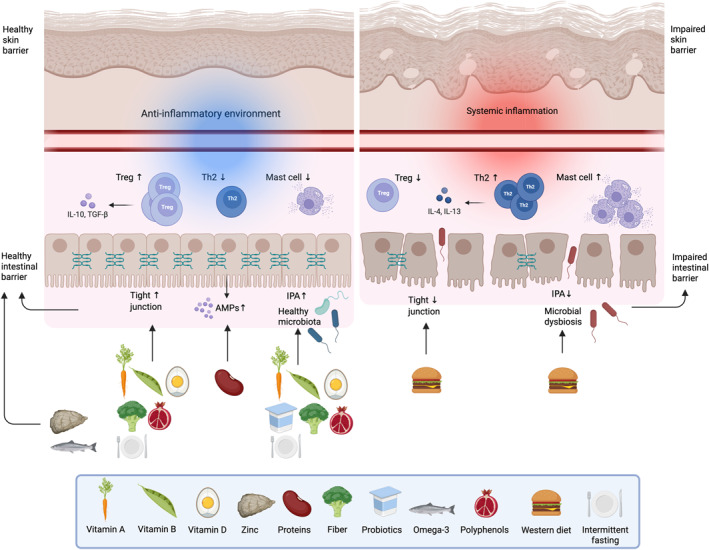
This illustration shows how dietary components and dietary patterns contribute to the function of the gut barrier. Vitamins A, B, and D, zinc, a high‐protein diet, fiber, omega‐3 fatty acids, probiotics, polyphenols and intermittent fasting enhance epithelial barrier integrity by promoting a healthy microbiota, stimulating the production of antimicrobial peptides (AMPs), encouraging goblet cells to produce mucus, supporting epithelial cells in forming tight junctions and increasing SCFAs and IPA levels. In contrast, a Western diet can compromise tight junctions and disrupt gut microbial communities, leading to dysbiosis and reduced SCFA and IPA levels. This weakens the gut mucus layer, allowing microbes, allergens, emulsifiers from processed foods, and detergents to penetrate the intestinal barrier. As a result, toxic metabolites are produced, immune cell activity is altered, and inflammation is triggered. These effects can spread through the circulatory system, ultimately contributing to skin impairment (Created in BioRender. Ryczaj, K. (2025) https://BioRender.com/tl9nhi3).

A growing body of evidence underscores the critical relationship between diet and skin barrier function. Nutrient deficiencies highlight the indispensable role of micro‐ and macronutrients, as well as other dietary constituents, in maintaining the structural and functional integrity of the skin. A nutritionally balanced diet, enriched with components that support keratinocyte differentiation, the cornified envelope, skin lipid synthesis, pH homeostasis, microbiome balance, collagen biosynthesis, and immune regulation, is paramount for preserving a resilient skin barrier. Conversely, minimizing intake of dietary factors detrimental to skin health is equally essential (Table 2, Figures 1 and 2).

**TABLE 2:** Summary of key findings on the roles of nutrients and dietary patterns in skin health.

Nutrient/Dietary pattern	Impact on skin health
Physical and biochemical barrier	
Vitamin C	— Antioxidant protection — Photoprotection — Collagen synthesis — Supports keratinocyte differentiation — Enhances ceramide synthesis — Supports wound healing
Vitamin E	— Antioxidant protection — Photoprotection — Collagen synthesis — Supports wound healing
Vitamin A	— Antioxidant protection — Photoprotection — Regulates keratinocyte differentiation and proliferation — Stimulates proteins forming the cornified envelope — Enhances tight junctions — Collagen synthesis — Promotes angiogenesis — Supports wound healing
Vitamins B	— Antioxidant protection — Photoprotection — Stimulates collagen synthesis — Enhances ceramide and lipid synthesis — Supports keratinocyte differentiation — Stimulates proteins forming the cornified envelope — Reduces TEWL — Supports wound healing
Vitamin D	— Photoprotection — Supports keratinocyte differentiation and proliferation — Enhances ceramide and lipid synthesis — Stimulates proteins forming the cornified envelope — Enhances tight junctions — Supports wound healing
Iron	— Stimulates collagen synthesis — Supports wound healing — Implicated in ROS production and photo‐induced skin damage
Zinc	— Antioxidant protection — Photoprotection — Stimulates collagen synthesis — Supports keratinocyte differentiation — Supports wound healing
Copper	— Antioxidant protection — Stimulates collagen synthesis — Supports wound healing
Selenium	— Antioxidant protection — Photoprotection — Supports keratinocyte differentiation — Supports wound healing
Proteins	— Collagen production — Supports wound healing
Carbohydrates	Fiber: — Antioxidant protection — Stimulate collagen synthesis — Supports wound healing
PUFAs	Omega‐3: — Antioxidant protection — Photoprotection — Slow collagen breakdown — Support keratinocyte differentiation — Increase ceramide levels — Reduce TEWL — Support wound healing, reduces redness and sensitivity Omega‐6: — Increase ceramide levels — Reduce TEWL — Support wound healing, reduces redness and sensitivity
MUFAs	— Antioxidant protection — Support wound healing
TFAs	— Increase the generation of ROS
Probiotics	— Antioxidant protection — Photoprotection — Collagen protection — Support keratinocyte differentiation — Reduces TEWL — Support wound healing, reduce erythema, skin sensitivity
Polyphenols	— Antioxidant protection — Photoprotection — Protect against cigarette smoke — Stimulate collagen synthesis — Support keratinocyte differentiation — Reduce TEWL — Support wound healing
Western diet	— Induces systemic oxidative stress — Affects protein function in the dermis — Modify lipid composition of the skin — Alters skin microbiota
Mediterranean diet	— Antioxidant effects — Promotes overall skin health
Plant‐based diet	— Antioxidant effects — Promotes overall skin health
Immune response	
Vitamin C	— Anti‐inflammatory properties
Vitamin E	— Anti‐inflammatory properties — Enhances Th1‐mediated immune responses, suppresses Th2‐mediated pathways — Antimicrobial properties
Vitamin A	— Anti‐inflammatory properties — Supports intestinal barriers that contribute to skin health
Vitamins B	— Anti‐inflammatory properties — Enhances Th1‐mediated immune responses, suppresses Th2‐ and Th17‐mediated pathways — Supports intestinal barriers that contribute to skin health
Vitamin D	— Anti‐inflammatory properties — Enhances Th1‐mediated immune responses, suppresses Th2‐mediated pathways — Antimicrobial properties — Supports intestinal barriers that contribute to skin health
Iron	— Deficiency linked to low‐grade inflammation and Th2‐skewed responses
Zinc	— Anti‐inflammatory properties — Enhancing Th1‐mediated immune responses — Zinc deficiency increased Th17 and Th2‐ mediated pathways — Supports intestinal barriers, contributing to skin health
Copper	— Antimicrobial properties
Selenium	— Anti‐inflammatory properties — Enhancing Th1‐mediated immune responses
Proteins	— Regulate adaptive and innate immune responses — High‐protein diet has anti‐inflammatory effects — Protein deficiency promotes pro‐inflammatory cytokines — Supports intestinal barriers, contributing to skin health
Carbohydrates	— Anti‐inflammatory effects in specific populations
PUFAs	Omega‐3: — Anti‐inflammatory properties — Enhances Th1‐mediated immune responses, suppresses Th2‐mediated pathways — Supports intestinal barriers, contributing to skin health. Omega‐6: — Pro‐ inflammatory properties
MUFAs	— Anti‐inflammatory properties
SFAs	— Pro‐ inflammatory properties — Promote Th2 and Th17 responses
TFAs	— Pro‐ inflammatory properties
Probiotics	— Anti‐inflammatory properties — Suppress Th2 and Th17 responses — Supports intestinal barriers that contribute to skin health
Postbiotics	— Anti‐inflammatory properties — Suppress Th2 response — Influence skin microbiota — Supports intestinal barriers that contribute to skin health
Polyphenols	— Anti‐inflammatory properties — Enhancing Th1‐mediated immune responses while suppressing Th2‐mediated pathways — Antimicrobial properties — Supports intestinal barriers, contributing to skin health
Western diet	— Pro‐inflammatory properties — Th2‐driven immune responses — Alterations in the gut microbiome, compromise of the gut epithelial barrier
Mediterranean diet	— Anti‐inflammatory properties
Plant‐based diet	— Anti‐inflammatory properties
Intermittent fasting	— Anti‐inflammatory properties — Suppress Th2 response — Supports intestinal barriers, contributing to skin health

Abbreviations: AQP3, aquaporin‐3; IPA, Indole‐3‐propionic acid; MUFAs, Monounsaturated fatty acids; NF‐κB, the nuclear factor kappa B; PUFAs, Polyunsaturated fatty acids; ROS, reactive oxygen species; SFAs, Saturated fats; TEWL, trans epidermal water loss; TFAs, Trans fatty acids; Treg, regulatory T cells.

In the context of modern urbanized environments, where external stressors exacerbate challenges to skin health, strategic dietary modifications emerge as a promising intervention. Nutritional modifications show promise in the prevention and management of AD. However, inconsistencies and gaps within the current scientific literature impede the development of definitive dietary guidelines for skin health and the management of AD.

Dietary supplements, widely marketed as solutions for nutritional deficiencies and as aids to contemporary lifestyles, warrant cautious use. Excessive consumption of these products may lead to nutrient toxicities and adverse effects. Therefore, while diet remains a powerful tool for supporting skin health, emphasis should be placed on achieving balance through whole foods rather than supplementation.

### 6.1 Future Directions

Although the link between diet and skin health is increasingly recognized, significant research gaps remain, particularly regarding the direct effects of nutrition on skin barrier function, independent of AD outcomes. Most available data come from observational studies or clinical trials assessing AD symptoms, making it difficult to isolate whether observed effects are due to direct improvement of the skin barrier or indirect modulation of immune or microbiome‐related pathways.

To advance the field, future studies should:

- Well‐designed interventional studies in healthy individuals or at‐risk populations, focusing on direct skin outcomes.
- Use validated, standardized tools for assessing dietary intake, including dietary diversity indices and biomarkers of nutritional status.
- Apply objective skin measurements such as TEWL, stratum corneum hydration, lipid composition analysis, and tape stripping for molecular analysis.
- Explore the interactions between nutrition, the microbiome, immune tolerance, and epithelial barrier function, using integrative approaches such as metabolomics, transcriptomics, and epigenetics.
- Include genetic and epigenetic factors, to better understand gene–diet interactions and personalize dietary recommendations.
- Design RCTs examining maternal diet during pregnancy, infant diet, and their combined effect on early skin development and barrier function.

## Author Contributions

**Klaudia Ryczaj:** conceptualization, investigation, writing – original draft, methodology, visualization, writing – review and editing. **Burcin Beken:** investigation, writing – original draft, methodology, writing – review and editing, supervision. **Cezmi Akdis:** writing – review and editing, supervision.

## Conflicts of Interest

The authors declare no conflicts of interest.

## Supporting information


Supporting Information S1

## Data Availability

Data sharing is not applicable to this article as no new data were created or analyzed in this study.
