# Supplementary material for: Feeding the Skin Barrier: The Impact of Macro‐ and Micronutrients on Skin Barrier Function
Source: Clin Transl Allergy. 2025 Nov 18;15(11):e70105. doi: 10.1002/clt2.70105 (PMC12626168; doi:10.1002/clt2.70105)
Supplement: Supplementary file 1 — Supporting Information S1 [file CLT2-15-e70105-s001.docx]

**Skin barrier in healthy skin**

The skin barrier is a complex and dynamic structure that serves as the primary interface between the body and the environment. It is composed of the epidermis, dermis, and subcutaneous fat, with the epidermis being the outermost layer responsible for barrier function. Together they form a robust, multilayered defense system that integrates physical, chemical, and immunological components, ensuring the skin's ability to protect against external insults while preserving internal equilibrium.

Key components of the skin barrier in healthy skin:(1,2)

1. Epidermis

- Corneocytes: Flattened, dead cells forming the "bricks" in the "brick and mortar" model of the skin barrier. They contain:
  - - Keratin filaments for structural support
    - Filaggrin (FLG), which aggregates keratin and helps form natural moisturizing factors (NMF)
- Intercellular lipids: The "mortar" between corneocytes, consisting of:
  - - Ceramides
    - Cholesterol
    - Free fatty acids
- Cornified cell envelope: A robust protein/lipid polymer structure composed of:
  - - Proteins: loricrin, involucrin, small proline-rich proteins (SPRPs)
    - Lipids: ceramides A and B covalently bound to proteins
- NMF: Hygroscopic molecules inside skin cells that attract and retain water, including amino acids, urea, and lactate, which maintain hydration and elasticity.
- Tight junctions (TJs): Located in the granular layer of the epidermis, consisting of proteins such as claudins and occludins, they regulate selective permeability and prevent harmful agent infiltration.
- Acid mantle: Maintains an acidic pH on the skin surface, optimizing enzymatic functions and protecting against pathogens.
- Microbiome: Commensal microbes contribute to immune defense, preventing pathogenic colonization and promoting skin homeostasis.

1. Dermis

- The dermis lies beneath the epidermis and provides structural support, strength, and elasticity to the skin. It is composed of fibroblasts that synthesize collagen and elastin, essential for maintaining skin integrity.
- This layer also houses vital structures such as nerve endings, vascular networks, sebaceous glands, and hair follicles, all of which contribute to skin barrier function by supporting hydration, thermoregulation, and immune responses.

**Skin Barrier in AD**

In AD, the skin barrier is compromised due to structural and functional abnormalities, leading to increased TEWL, reduced hydration, and heightened susceptibility to allergens and pathogens.

Key Alterations in AD:(3,4)

- Corneocyte defects:
  - Impaired keratinocyte differentiation results in defective corneocyte aggregation.
  - Reduced expression FLG leads to lower NMF levels and increased dryness.
- Lipid composition changes:
  - Decreased ceramide levels and shorter free fatty acid chains weaken the extracellular lipid matrix, impairing barrier integrity.
  - The compromised barrier results in increased TEWL, leaving the skin dry and prone to external insults.
- Compromised cornified cell envelope:
  - Altered protein and lipid composition weakens the protective layer, increasing permeability to irritants and allergens.
  - Reduced expression of key structural proteins such as involucrin, loricrin, and claudin contributes to barrier dysfunction.
- Disrupted TJs:
  - Dysregulation of junctional proteins such as claudins and occludins leads to increased permeability, allowing allergens and microbes to penetrate deeper layers of the skin.
- Acid mantle disruption:
  - Reduced FLG catabolism exacerbates the lack of acidity required for lipid-processing enzymes, further weakening the barrier.
- Microbial dysbiosis:
  - Increased colonization by *Staphylococcus aureus* exacerbates inflammation and skin barrier damage.
  - Loss of beneficial microbes weakens immune defenses and promotes flare-ups.
- Immune dysregulation:
  - Damaged keratinocytes release epidermal alarmins (IL-33, IL-25, TSLP), which activate immune pathways and sustain inflammation.
  - Persistent type 2 cytokine activity (IL-4, IL-13) suppresses barrier repair mechanisms, worsening the dysfunction.

**NUTRIENTS**

**1.MICRONUTRIENTS**

**1.1 VITAMINS**

**VITAMIN C**

Vitamin C concentration in the skin is closely linked to various biological functions essential for skin health:

1. Antioxidant protection and photoprotection

As a potent antioxidant, it defends against reactive oxygen species (ROS) generated by environmental stressors, including pollutants and ultraviolet (UV) radiation. By neutralizing free radicals, chelating metal ions, and mitigating oxidative damage, vitamin C helps to preserve the integrity of the skin barrier. Oxidative stress can impair this barrier by inducing lipid peroxidation and promoting inflammatory responses.(5) Although the skin possesses endogenous antioxidant defenses, prolonged environmental exposure can deplete these protective reserves, weakening the skin's ability to resist oxidative damage. Furthermore, a combination of vitamins E and C exhibits synergistic effects, enhancing photoprotection, reducing sunburn severity, and mitigating UV-induced skin damage.(2,6)

2. Collagen production

Vitamin C also plays a pivotal role in collagen synthesis, which is essential for maintaining skin strength and structural integrity. As a cofactor for prolyl and lysyl hydroxylase, it stabilizes the triple-helical structure of collagen. Additionally, it promotes collagen gene expression and stimulates fibroblast activity, thereby maintaining the extracellular matrix.(7–9)

3. Keratinocyte differentiation

Beyond its role in collagen metabolism, vitamin C supports keratinocyte differentiation, key processes in preserving the skin's barrier function.(8,9)

4. Lipid Synthesis

Vitamin C enhances ceramide synthesis, which contributes to the maintenance of a healthy and resilient skin barrier.(8,9)

5. Role in immune regulation

The vitamin’s C anti-inflammatory properties further contribute to skin health. It suppresses activation of the nuclear factor kappa B (NF-κB) pathway, thereby reducing the production of proinflammatory cytokines.(10) Studies indicate that vitamin C reduces levels of cytokines such as tumor necrosis factor-alpha (TNF-α) and interferon-gamma (IFN-γ) while increasing anti-inflammatory cytokine interleukin-10 (IL-10).(11) Moreover, it decreases histamine levels, a known inducer of pruritus, which is particularly relevant in AD.(10)

6. Wound Healing

Vitamin C is crucial for wound healing, primarily due to its role in enhancing collagen production and supporting tissue repair.(7,8)

Symptoms of deficiency

Clinically, vitamin C deficiency manifests in conditions such as scurvy, characterized by symptoms including skin fragility, petechial lesions, gum bleeding, easy bruising, and delayed wound healing—highlighting the vitamin's essential role in maintaining skin integrity.(2,12)

**VITAMIN E**

Vitamin E plays an important role in skin health:

1. Antioxidant protection and photoprotection

α -Tocopherol is considered to be the most potent antioxidant. It plays a critical role in photoprotection, safeguarding the skin from UV-induced damage. When combined with vitamin C, α-tocopherol’s antioxidant and photoprotective properties are significantly enhanced, offering greater defense against oxidative stress.(2,5,6)

2. Collagen production

Additionally, vitamin E takes part in biosynthesis of collagen.(13)

3. Role in immune regulation

Vitamin E exerts immunomodulatory effects by enhancing Th1-mediated immune responses while suppressing Th2-mediated pathways.(8) Vitamin E has been suggested to exhibit anti-inflammatory activity by downregulating NF-κB kinase.(14) Furthermore, vitamin E has been shown to downregulate pathways involved in the production of eicosanoids like leukotrienes and prostaglandin E2 (PGE2).(15) Reduced PGE2 levels have been linked to decreased IgE production, which is integral to allergic responses.(16)

4. Antimicrobial effects

Vitamin E also exhibits antimicrobial properties, with studies showing it reduces microbial adhesion, including *Staphylococcus epidermidis* and *Staphylococcus aureus*,(17) pathogens often implicated in skin barrier disruption and AD flares.(18)

5. Wound healing

Vitamin E supports wound healing.(2,13)

Symptoms of deficiency

Deficiency in vitamin E can result in a variety of dermatological manifestations, including skin dryness, edema with papular erythema, depigmentation, and seborrheic changes.(2,12) These effects are particularly pronounced in premature infants, highlighting the vitamin’s essential role in maintaining skin integrity.(19) However, a cross-sectional study have not established a direct correlation between vitamin E intake and improvements in skin hydration.(20)

**VITAMIN A**

Vitamin A contributes significantly to skin health:

1. Antioxidant protection and photoprotection

Vitamin A and its derivatives play a pivotal role in protecting the skin from environmental stressors, such as UV radiation and oxidative damage. Retinoids act as potent antioxidants, neutralizing ROS, preserving lipid integrity, and mitigating inflammation.(2,12) Studies have demonstrated that vitamin A supplementation reduces UV-induced skin damage, minimizes erythema, and prevents moisture loss in irradiated skin.(12,19)

2. Collagen production

Retinol also promotes fibroblast activity, collagen synthesis, processes crucial for maintaining skin strength and elasticity.(12,21)

3. Keratinocyte differentiation

A key function of vitamin A in skin health is its regulation of keratinocyte differentiation and proliferation. Retinoic acid (RA), the active metabolite of vitamin A, binds to nuclear receptors in keratinocytes, influencing gene expression critical to their maturation.(12,19)

4. Formation of the cornified envelope

Retinoids also stimulate the production of proteins necessary for forming the cornified envelope, a critical component of the skin barrier.(21)

5. TJs

Additionally, vitamin A enhances the formation of TJs in keratinocyte cultures and regulates TJ protein expression.(22)

6. Role in immune regulation

Vitamin A exhibits notable immunomodulatory effects, shaping inflammatory pathways and allergic responses through various mechanisms. RA has been shown to inhibit allergic reactions by preventing mesenteric lymph node dendritic cells (MLN DCs) from inducing IL-13-producing inflammatory Th2 cells.(23) Additionally, RA supports immune balance by promoting the differentiation of regulatory T cells (Treg)(24) which promote the regeneration of epithelial stem cell and production of anti-inflammatory cytokine IL-10.(2,25) Vitamin A also modulates B cell differentiation, influencing the secretion and synthesis of IgE antibodies.(26) Vitamin A are also involve in inhibition of NF-k.(10,11)

These findings, however, are at odds with other studies that have reported vitamin A supplementation to promote allergic responses. The discrepancies regarding the role of RA in Th2 cell differentiation may be attributed to variations in the doses of biologically available RA utilized across different studies. Interestingly, the influence of RA on Th17 cell development appears dose-dependent, as physiological concentrations stimulate their differentiation, whereas higher doses inhibit this process. These findings suggest that maintaining sufficient vitamin A levels may be required for controlling allergic diseases and achieving immune homeostasis.(27,28)

7. Role in gut-skin axis

Vitamin A supports the intestinal microbiota and enhances the expression of TJ proteins, thereby strengthening the gut barrier and contributing to the integrity of the skin barrier.(8,29) (Figure 2)

8. Wound healing

In addition to its role in collagen production, retinol supports angiogenesis all of which are integral to effective wound healing.(12,21)

Symptoms of deficiency and overdosing

Deficiency in vitamin A can lead to a range of dermatological manifestations, including follicular papules with keratin plugs, commonly found on the extensor surfaces, buttocks, and shoulders. As deficiency progresses, these lesions can enlarge and spread to the trunk and face. Other associated findings include generalized xerosis, desquamation, and violaceous-brown macules in areas of hyperkeratosis.(7)

While vitamin A supplementation offers therapeutic benefits for skin health, it is important to note that high doses, especially of certain forms like retinyl palmitate, may have adverse effects, such as an increased risk of lung cancer.(12)

**VITAMIN B**

Vitamin B plays an important role in skin health:

1. Antioxidant protection and photoprotection

B vitamins help shield the skin from oxidative stress and contributes to the skin's resilience against environmental stressors such as UV radiation and pollution.(30)

2. Collagen production

Niacinamide, the amide form of niacin, stimulates fibroblast activity, promoting the synthesis of collagen.(31,32) Pyridoxine facilitates collagen formation by supporting proline availability, an amino acid essential for stabilizing collagen structures.(31)

3. Keratinocyte differentiation

In addition, niacinamide accelerates keratinocyte differentiation and upregulates the expression of FLG.(31,32)

4. Formation of the cornified envelope

Niacinamide upregulates the expression of involucrin, which are integral to the formation of the cornified envelope.(31–33)

5. Lipids synthesis

Niacinamide enhances the synthesis of key stratum corneum lipids, including ceramides, sphingomyelin, fatty acids, and cholesterol, which are essential for barrier integrity.(32)

6. Modulation of aquaporin-3 (AQP3) expression

Research shows that niacinamide modulates aquaporin-3 (AQP3) expression. Elevated AQP3 levels, commonly observed in AD lesions, are linked to reduced FLG expression and increased inflammatory markers.(34)

7. Role in immune regulation

Vitamins B exhibits anti-inflammatory properties, primarily by inhibiting the NF-κB signaling pathway.(30) Pyridoxine, specifically, plays a role in modulating immune responses by supporting Th1-mediated activity while suppressing Th2 cytokine-driven pathways, thereby maintaining a balanced inflammatory response.(8)

Folic acid has also been shown to possess significant anti-inflammatory properties, particularly in allergic conditions such as AD. Evidence from animal models of AD indicates that high-dose oral folic acid supplementation effectively reduces inflammation by suppressing T-cell proliferation and decreasing Th2-and Th17-related cytokines, and IL-33 and TSLP.(35) Additionally, folic acid supports Treg cell survival, further contributing to immune regulation. These findings highlight folic acid's potential role in modulating skin inflammation.

However, niacin, by activating receptors in dermal Langerhans cells leading to the release of prostaglandins in the surrounding capillaries. This can result in adverse effects such as erythema, stinging, or itching.(36)

8. Role in gut-skin axis

B-group vitamins support the intestinal microbiota and enhances the gut barrier.(8) For example, they have been found to increase levels of *Faecalibacterium prausnitzii*, a key butyrate-producing bacterium with anti-inflammatory properties, while reducing populations of *Escherichia coli*. In addition to being obtained from the diet, many B vitamins are synthesized by gut microbes such as *Bifidobacterium* or *Lactobacillus*.(37,38) These vitamins can also be exchanged between microbes via cross-feeding, a process essential for maintaining microbial community stability.(38) Moreover, dietary fiber enhances the microbial synthesis of B vitamins in the colon.(39) Emerging evidence suggests that B-group vitamins may function as modulators, and potentially as prebiotic candidates, within the gut-skin axis.(38)

9. Hydration

Niacinamide can reduces TEWL, contributing to improved skin hydration.(40)

10. Wound healing

Clinical evidence underscores that B vitamin supplementation enhances wound healing, further demonstrating their pivotal role in skin repair.(31)

Symptoms of deficiency and overdosing

B vitamin deficiencies often manifest with similar dermatological symptoms, such as glossitis, xerosis, intertriginous red erosive lesions, and dermatitis. Severe niacin deficiency, as seen in pellagra, compromises the skin barrier, resulting in erythema, hyperpigmentation, and ulcerations in sun-exposed areas. Thiamine deficiency, associated with beriberi, can cause waxy, edematous skin.(31)

Conversely, excessive intake of B vitamins, can cause adverse reactions like skin flushing, pruritus, and irritation.(32)

**VITAMIN D**

Vitamin D plays an important role in skin health:

1. Photoprotection

Vitamin D protects keratinocytes from UVB-induced damage and apoptosis.(41)

2. Keratinocyte differentiation

Vitamin D supports keratinocytes differentiation and proliferation and enhances FLG production.(41,42)

3. Formation of the cornified envelope

By enhancing the production of structural proteins such as involucrin and loricrin, vitamin D contributes to maintaining the cornified envelope, a critical component of the skin barrier.(42)

4. TJs

Vitamin D boosts the expression of TJ proteins, thereby reinforcing epidermal integrity.(43)

5. Lipids synthesis

Vitamin D also regulates the production of ceramides and other epidermal lipids crucial for barrier function.(44) Studies using vitamin D receptor-deficient mice demonstrate that disrupted lipid transport due to reduced lamellar bodies impairs skin permeability and barrier homeostasis.(45)

6. Role in immune regulation

Vitamin D also exerts significant immunomodulatory effects. It enhances innate immunity by increasing antimicrobial peptides (AMPs) such as cathelicidin and defensins, which provide a frontline defense against pathogens. In adaptive immunity, vitamin D suppresses Th2 responses and promotes Th1 activity.(10) Additionally, it downregulates TLRs and IL-17,(46,47) inhibits prostaglandin production, and reduces IgE production by B cells.(48) Vitamin D also enhances the number of Treg cells and exerts anti-inflammatory effects by reducing excessive TNF-α production.(8,11,49)

7. Role in gut-skin axis

Additionally, vitamin D contributes to gut health by regulating the gut microbiome and enhancing TJs.(8)

8. Wound healing

Vitamin D supports wound healing by promoting keratinocyte proliferation, fibroblast activity, collagen synthesis, and the production of AMPs, which promote angiogenesis and regulate inflammation. (50,51)

**1.2 MINERALS**

**IRON**

Iron is crucial for maintaining skin health:

1. Role in oxidative stress and photo-induced skin damage

Under oxidative stress conditions, iron is released from its protein-bound form, catalyzing the production of ROS, which may exacerbate skin damage. Additionally, iron contributes to photo-induced skin damage, amplifying the harmful effects of UV radiation on the skin.(52)

2. Collagen production

In the context of skin health, iron contributes to collagen synthesis.(52)

3. Keratinocyte differentiation

Iron plays a crucial role in the differentiation and growth of epithelial tissues, supporting the maintenance of healthy skin and its capacity to renew and repair.(8)

4. Role in immune regulation

Iron also plays a pivotal role in immune regulation. Deficiency in iron is associated with low-grade inflammation and a pro-inflammatory shift, particularly in infants and children. Limited iron availability favors Th2 survival, promotes antibody class switching, and primes mast cells for degranulation.(28,53,54) Furthermore, low maternal hemoglobin levels during pregnancy have been linked to elevated IgE levels in offspring,(55) a finding that aligns with the higher IgE concentrations commonly observed in anemic patients.(56)

5. Wound healing

Iron is also integral to wound healing, highlighting its importance in tissue repair.(52)

Symptoms of deficiency

Dermatologically, iron deficiency manifests as pale skin, pruritus, increased susceptibility to infections, and angular cheilitis (inflammation at the corners of the mouth).(52)

**ZINC**

Zinc is fundamental to skin health:

1. Antioxidant protection and photoprotection

Zinc possesses antioxidant properties that protect skin cells from oxidative stress and photodamage by absorbing and reflecting UV radiation.(57)

2. Collagen production

Zinc plays a crucial role in collagen synthesis, which is vital for maintaining skin structure and elasticity.(58)

3. Keratinocyte differentiation

Zinc is integral to keratinocyte proliferation and differentiation, essential processes for maintaining healthy skin.(59) Zinc regulates the production of FLG, a protein critical for skin barrier integrity. It promotes FLG synthesis while inhibiting its metabolic breakdown, facilitating the conversion of FLG into urocanic acid (UCA). This conversion aids in skin acidification and strengthens immune defenses by modulating the local immune environment.(58,60,61)

4. Role in immune regulation

Zinc plays a multifaceted role in immune regulation. It inhibits the activation of the transcription factor NF-κB, thereby reducing the production of key pro-inflammatory cytokines and suppressing TLR4 signaling.(62,63) Zinc also modulates the differentiation of Th17 and Th9 cells, with deficiencies linked to increased Th17 production, contributing to a pro-inflammatory state.(8,10,11,64) Additionally, zinc is vital for preserving immune equilibrium by supporting Th1 responses. A deficiency in zinc disrupts this balance, promoting a Th2-skewed immune response, a hallmark of AD pathogenesis.(54,64) Zinc administration has been shown to increase the population of Tregs, promoting immune tolerance and mitigating excessive inflammation.(11) Additionally, zinc enhances macrophage phagocytic activity, strengthening the immune response against pathogens such as *Escherichia coli* and *Staphylococcus aureus.*(65)

5. Role in gut-skin axis

Zinc deficiency increases intestinal permeability.(66)

6. Wound healing

Zinc is also pivotal in wound healing.(7,58,67)

Symptoms of deficiency

Clinically, zinc deficiency presents with sharply demarcated, pink, scaly, eczematous plaques on the extremities and periorificial regions, often accompanied by impaired wound healing and reduced immune function.(7)

**COPPER**

This trace mineral plays a multifaceted role in maintaining skin health and integrity:

1. Antioxidant protection

Copper serves as a cofactor for antioxidant enzymes, contributing to the neutralization of free radicals and protecting the skin from oxidative stress.(8,68)

2. Collagen production

Copper plays a role in collagen synthesis by stimulating fibroblast proliferation and enhancing the production of key structural proteins, including collagen types I, II, and V, elastin, and fibrillin.(69)

3. Antimicrobial effects

Copper exhibits antibacterial properties against skin pathogens, including methicillin-resistant *Staphylococcus aureus* (MRSA).(69,70)

4. Wound healing

Copper is also essentially in all stages of the wound healing process and plays a key role in skin regeneration and angiogenesis.(69)

Symptoms of deficiency

Clinical manifestations of copper deficiency include impaired wound healing, hypopigmentation, alopecia, and seborrheic dermatitis.(7)

**SELENIUM**

Selenium is a vital trace element predominantly sourced from seafood, meats, nuts, and selenium-enriched wheat, depending on soil content. Absorption of selenium can be enhanced by concurrent intake of vitamins C and E.(5) Because selenium cannot be synthesized by the body, it must be obtained through diet or supplementation.(71)

Selenium plays an important role in skin health:

1. Antioxidant protection and photoprotection

Selenium is crucial for the functionality of selenoproteins, a class of proteins that play a central role in antioxidant defenses.(5,72) Selenium deficiency has been shown to increase susceptibility to UV-B radiation in mice, indicating reduced antioxidative defense against UV-induced damage.(73)

2. Keratinocyte differentiation

Selenium is essential for the growth, viability and differentiation of keratinocytes. It also supports keratinocyte adhesion to the basement membrane, reinforcing the structural integrity of the epidermis.(71,72,74)

3. Role in immune regulation

Adequate selenium levels are essential for regulating the immune response and preventing chronic inflammation.(49) Selenium supports immune function by enhancing Th1-cell activity and promoting T-cell stimulation.(11) Furthermore, selenium supplementation has been shown to suppress the gene expression of IL-1 and TNF-α, highlighting its anti-inflammatory potential.(75) In addition, selenium contributes to the body’s defense against pathogens.(72)

4. Wound healing

Selenium is a nutrient that promotes wound healing by promoting tissue repair and regeneration.(7)

Symptoms of deficiency

Clinically, selenium deficiency can manifest in dermatologic symptoms, such as changes in pigmentation, xerosis, and nail abnormalities. Deficiency is also linked to increased vulnerability to infections.(7)

**2. MACRONUTRIENTS**

**2.1 PROTEINS**

1. Collagen production

Protein intake is crucial for collagen synthesis. Studies have shown that maternal low-protein diets during lactation have been linked to decreased production of type I and III tropocollagen in animal models.(76)

2. Role in immune regulation

Amino acids, the building blocks of proteins, are integral to immune function. They regulate the activation of both adaptive and innate immune cells, including B cells, T cells, natural killer cells, and macrophages, and are crucial for lymphocyte proliferation as well as the synthesis of antibodies, cytokines, and cytotoxic molecules.(77)

Studies have shown that high-protein diets, regardless of whether the source is animal- or plant-based, reduce levels of proinflammatory adipokines such as chemerin and progranulin.(78) In contrast, protein deficiency leads to reduced amino acid availability, inducing cellular stress and activating T cells that release proinflammatory cytokines.(79)

3. Role in gut-skin axis

Furthermore, high-protein diets have been shown to enhance intestinal immunity by promoting the expression of AMPs,(80) and are linked to greater microbial diversity.(37) Pea protein intake increases beneficial Bifidobacterium and Lactobacillus, reduces harmful species like B. fragilis and C. perfringens, and boosts SCFA levels, supporting gut and immune health.(37)

4. Role in tissue construction and wound healing

Proteins are indispensable for the structure and function of tissues and organs, playing a critical role in tissue construction, repair, physiological regulation, and energy provision. The continuous renewal of cells within body tissues relies on adequate protein intake to ensure proper regeneration and repair. Research indicates that sufficient protein intake can promote healing, whereas both protein deficiency and excess can impair the healing process.(81)

**2.2 CARBOHYDRATES**

1. Antioxidant protection

Prebiotics have been associated with a reduction in oxidative stress.(84)

2. Collagen production

Prebiotics contribute to improved skin integrity and enhanced collagen production.(2)

3. Role in immune regulation

Research has demonstrated the anti-inflammatory effects of carbohydrate intake in specific populations. One study found that consumption of a carbohydrate-rich beverage resulted in a reduction of TLR4 receptor expression in the monocytes of obese/overweight children.(82) Another study in athletes showed that carbohydrate consumption was associated with a more balanced distribution of immune cells in the blood. This was accompanied by a reduction in the phagocytic activity of monocytes and granulocytes, as well as lower levels of ROS and inflammatory cytokines, further supporting the immunomodulatory role of carbohydrates.(83)

4. Role in gut-skin axis

Prebiotics play a crucial role in maintaining the balance of gut microbiota which is pivotal to the gut-skin axis. When intestinal barriers are compromised, intestinal bacteria and their metabolites have been observed to enter the bloodstream, accumulate in the skin, and disrupt skin homeostasis. Prebiotics restore intestinal microflora, maintain TJs, protect against pathogenic microorganisms, modulate the immune system, and repair intestinal barrier functions, providing significant benefits for the skin. (85)

5. Hydration

Prebiotics also contribute to increased hydration.(86)

6. Wound healing

Prebiotics support wound healing processes.(2,84,86,87)

**2.3 FATS**

**POLYUNSATURATED FATTY ACIDS (PUFAs)**

1. Antioxidant protection and photoprotection

Omega-3 fatty acids exhibit antioxidant properties, modulating oxidative stress pathways and protecting skin from damage, including UV-induced photodamage. (88)

2. Slowing collagen breakdown

Omega-3 fatty acids inhibit matrix metalloproteinase-1 (MMP-1) expression, which slows collagen breakdown, provide photoprotection and supports dermal structure.(88)

3. Keratinocyte differentiation

Omega-3 fatty acids promote keratinocyte differentiation and enhance FLG expression. (33,89)

4. Lipids synthesis

LA plays a structural role in the epidermis as a precursor for ceramides, which are crucial for building and maintaining the stratum corneum barrier.(33) Omega-3 increases ceramide levels, further contributing to skin barrier integrity.(90)

5. Role in immune regulation

Omega-3 fatty acids modulate inflammation by competing with omega-6 fatty acids for enzymatic pathways, reducing the synthesis of pro-inflammatory mediators such as prostaglandins and leukotrienes.(91,92) EPA and DHA lower the activation of inflammatory transcription factors like NF-κB, and reduce proinflammatory cytokine levels as well as IL-4 and IL-13.(33,93–95) DHA suppresses Th2 responses, enhances Th1 activity, and supports Treg development, promoting immune balance critical in AD.(96,97) Additionally decreases IgE production, which plays a role in allergic reactions.(16) Omega-3 fatty acids can reduce inflammation from TLR-2 activation, which is frequently triggered by *Staphylococcus aureus* colonization, a common issue in AD patients.(33)

Despite their benefits, omega-6 metabolites are vulnerable to oxidative stress from UV radiation, pollutants, and ozone, which can activate pro-inflammatory pathways involving eicosanoids such as thromboxane A2, prostaglandin E2 (PGE2), and leukotriene B4. These compounds contribute to skin inflammation and immune dysregulation.(91) PGE2 has been implicated in stimulating IgE production, a key driver of allergic and inflammatory processes.(16) Additionally, omega-6 fatty acids have been linked to increased production of TNF, further amplifying inflammatory responses.(98) In contrast, GLA stands out for its anti-inflammatory properties. GLA has been shown to enhance ceramide synthesis and promote epidermal cell proliferation, supporting barrier repair and skin regeneration.(99)

6. Role in gut-skin axis

Omega-3 fatty acids enhance intestinal barrier integrity.(100) Moreover, intake of n-3 polyunsaturated fats has been linked to stable or positive modulation of the gut microbiota, including higher levels of beneficial bacteria like Bifidobacterium, Lactobacillus, Streptococcus, and Akkermansia muciniphila.(37)

7. Hydration

Omega-3 and omega-6 fatty acids may help improve hydration and reduce TEWL.(99,101,102)

8. Wound healing

Omega-6 and omega-3 fatty acids have also been associated with enhanced skin barrier repair and wound healing.(88,103)

9. Reduction of Erythema and Skin Sensitivity

Omega-3 fatty acids may help mitigate inflammatory responses to skin irritants, reducing redness and sensitivity.(99,101,102) Omega-6 fatty acids contribute to improved skin texture by decreasing roughness and scaling.(99)

**MONOUNSATURATED FATTY ACIDS (MUFAs)**

MUFAs, primarily represented by oleic acid, are abundant in sources such as olive oil, avocados, and various nuts. MUFAs contributes significantly to skin health:

1. Antioxidant protection

MUFAs demonstrate antioxidant properties, contributing to their potential role in reducing oxidative stress and supporting skin health.(104,105)

2. Role in immune regulation

MUFAs also play a role in immune regulation by modulating inflammatory pathways.(104,105) Research highlights that olive oil can reduce the expression of NF-κB, a key regulator of inflammation.(104) Additionally, olive oil has been associated with decreased neutrophil elastase levels, an enzyme implicated in chronic inflammation and tissue degradation.(105)

3. Wound healing

MUFAs contribute to wound healing by promoting re-epithelialization, enhancing collagen deposition, and supporting angiogenesis, all of which are essential for effective tissue repair.(104–106)

**SATURATED FATS (SFAs)**

1. Role in oxidative stress

SFAs are known to increase ROS production and impair antioxidant defences, thereby promoting oxidative stress and contributing to tissue damage and inflammatory responses.(107)

2. Role in immune regulation

Emerging evidence underscores the pro-inflammatory potential of SFAs. High dietary intake of saturated fats can activate TLRs on immune cells, triggering the release of pro-inflammatory cytokines and chemokines. This process is associated with increased production of cytokines such as TNF-α, IL-6, and IL-1β, all of which play a critical role in the pathogenesis of chronic inflammatory diseases. Additionally, SFAs have been linked to the activation of type 3 innate lymphoid cells (ILC3s) and subsequent production of cytokines such as IL-17 and IL-22. These cytokines are known to exacerbate inflammatory conditions, including AD.(108,109) Saturated fat consumption has also been shown to induce the production of TSLP, IL-25, and IL-33, cytokines that promote Th2 responses. Such responses are characteristic of allergic diseases, including AD, highlighting the potential role of SFAs in immune dysregulation.(110)

3. Role in gut-skin axis

Diets high in saturated fats have been associated with an increase in Bilophila and Faecalibacterium prausnitzii populations, alongside a reduction in beneficial Bifidobacterium, potentially altering gut microbial balance.(37)

**TRANS FATTY ACIDS (TFAs)**

1. Role in oxidative stress

TFAs contribute significantly to oxidative stress and increase the generation of ROS.(111)

2. Role in immune regulation

TFAs activate TLRs, key mediators of innate immune responses. (112) Studies have demonstrated a positive correlation between TFAs intake and elevated plasma levels of inflammatory biomarkers, including IL-6, and E-selectin.(113) These markers serve as indicators of systemic inflammation and are closely linked to chronic inflammatory conditions.

3. Role in gut-skin axis

Diets rich in industrial TFAs have been shown to disrupt microbial balance by promoting the growth of potentially pathogenic bacteria such as Desulfovibrionaceae and Proteobacteria, while simultaneously reducing the abundance of beneficial taxa like Bacteroidetes, Lachnospiraceae, and Bacteroidales.(114)

**3. OTHER COMPOUNDS**

**3.1 PROBIOTICS AND POSTBIOTICS**

**PROBIOTICS**

1. Antioxidant protection and photoprotection

Consumption of fermented dairy products, abundant in probiotics and prebiotics, have been associated with a reduction in oxidative stress.(115–117) Additional benefits of probiotics include protection against UV radiation.(87)

2. Collagen protection

Probiotics inhibit the activity of MMPs, enzymes responsible for degrading dermal collagen.(87)

3. Keratinocyte differentiation

Fermented dairy products have been shown to promote keratinocyte differentiation. (115–117)

4. Role in immune regulation

Probiotics exhibit robust anti-inflammatory properties by modulating systemic and local immune responses. They suppress mast cell-mediated inflammation, reducing mast cell count, IgE levels, and IL-4 and TSLP production, while downregulating the expression of pro-inflammatory cytokines such as TNF-α, IL-6, and those associated with the IL-23/IL-17A axis.(87) Moreover, probiotics increase circulating levels of TGF-β, a cytokine known to support barrier integrity and reduce skin sensitivity.(87)

5. Role in gut-skin axis

Probiotics, as well as prebiotics, play an essential role in maintaining the balance of gut microbiota which is pivotal to the gut-skin axis. Probiotics restore intestinal microflora, protect against pathogenic microorganisms, modulate the immune system, and repair intestinal barrier functions, yielding significant benefits for the skin.(114)

6. Hydration

Probiotics promoting hydration and decreasing TEWL.(87,117) A meta-analysis evaluating the effects of lactic acid bacteria on skin moisturizing revealed varied outcomes depending on the formulation. When administered as a single formulation, lactic acid bacteria showed no significant improvement in skin hydration compared to placebo. However, when combined with complementary ingredients such as honeybush extract or GOS, the formulation exhibited statistically significant enhancements in skin hydration and reductions in TEWL, highlighting the potential of multi-component probiotic interventions.(118)

7. Wound healing

Probiotics enhance dermal thickness, improve cutaneous blood flow, and accelerate wound healing.(87)

8. Reduction of Erythema and Skin Sensitivity

Probiotics reduce erythema, skin sensitivity, and inflammation.(87,117)

**POSTBIOTICS**

Postbiotics are bioactive compounds made when the probiotic bacteria digest and break down prebiotics. These include short-chain fatty acids (SCFAs) such as acetate, propionate, and butyrate. Indole-3-propionic acid (IPA), a tryptophan-derived gut bacteria (Clostridium sporogenes) product, participates in intestinal barrier function.(119) Dietary sources of tryptophan include milk, cheese, egg white, chicken, fish, peanuts, soy beans, sesame, pumpkin seeds, and sunflower seeds. Diet plays an important role in regulating the microbiota-tryptophan axis, as high-fat and ketogenic diets lower IPA production, whereas a high-fiber diet promotes its synthesis.(120)

1. Lipids synthesis

SCFAs are hypothesized to play a role in enhancing skin ceramide synthesis.(86)

2. Acidifying the skin's pH

Moreover, SCFAs are hypothesized to contribute to the acidification of the skin's pH.(86)

3. Role in immune regulation

SCFAs have been shown to suppress inflammation by inhibiting histone deacetylase activity and NF-κB signaling pathways, which are central to inflammatory processes. Furthermore, butyrate stimulates the accumulation of Tregs and the production of key cytokines such as IL-10 and TGF-β, thereby enhancing immune tolerance and reducing systemic inflammation.(87,121) Additionally, SCFAs decrease the levels of IgE and TSLP.(122) SCFAs are also thought to play a crucial role in shaping specific skin microbiome profiles, which in turn influence the immune defense mechanisms of the skin. These mechanisms collectively contribute to skin homeostasis by mitigating inflammatory responses and supporting the integrity of the skin barrier.(2,87,123)

IPA alleviates LPS-induced inflammatory injury in human colonic epithelial cells by inhibiting LPS-induced intestinal epithelial cell apoptosis and inhibit the release of pro-inflammatory cytokines such as IL-1β, IL-6 and TNF-α via regulation of the Toll-like receptor 4 (TLR4)/myeloid differentiation factor 88/NF-κB and TLR4/TRIF/NF-κB pathways.(124)

4. Role in gut-skin axis

IPA has been found to increase transepithelial electrical resistance and decrease cellular permeability by increasing TJ proteins such as claudin-1, occludin, and ZO-.(124,125) IPA also has a strengthening effect on mucus barrier by increasing mucins and goblet cell secretions.(125)

**1.3.2 POLYPHENOLS**

Polyphenols exhibit a wide range of beneficial effects on skin health:

1. Antioxidant protection and photoprotection

Polyphenols are noted for their potent antioxidant properties, which support skin integrity and defence mechanisms against oxidative stress. These antioxidant effects enhance skin resilience to environmental stressors, including UV radiation, with studies indicating that polyphenols can delay the onset of UV-induced erythema.(126)

**2. Protection Against Environmental Damage**

Polyphenols also exhibit protective effects against environmental damage, such as cigarette smoke-induced loss of SR-B1, a cholesterol receptor critical for keratinocyte function.(127)

3. Collagen production

Polyphenols play a crucial role in preserving collagen by inhibiting matrix metalloproteinases (MMPs), which degrade collagen, while simultaneously promoting its synthesis.(128)

4. Keratinocyte differentiation

They also contribute to maintaining skin structure and function by promoting keratinocyte differentiation and regulating excessive proliferation.(128)

5. Role in immune regulation

Polyphenols exhibit strong anti-inflammatory properties and help modulate immune responses. They regulate the Th1/Th2 balance, stabilize mast cell membranes to reduce histamine release, and downregulate IgE secretion, potentially mitigating allergic responses and inflammation.(126,129,130) Additionally, polyphenols suppress inflammation by modulating key signaling pathways, such as NF-κB and may promoting anti-inflammatory mediators like IL-10.(129,131,132) At the same time, they inhibit Th2 cytokines as well as IL-25, IL-33 and TSLP.(129,131)

6. Antimicrobial effects

Polyphenols exhibit antimicrobial properties, targeting skin pathogens like *Staphylococcus aureus* and ceramidase-producing *Pseudomonas aeruginosa*, which can reduce ceramide levels and exacerbate skin conditions.(126)

7. Role in gut-skin axis

Polyphenols promote the expression of TJ proteins, modulate the gut microbiota, and enhance gut epithelial barrier function.(133,134) They also stimulate the growth of beneficial bacteria such as Bifidobacterium and Lactobacillus, which in turn enhances the production of SCFAs, metabolites essential for maintaining intestinal homeostasis and supporting immune regulation.(37)

8. Hydration

A meta-analysis confirmed their effectiveness of polyphenols in improving stratum corneum hydration and reducing TEWL, though further research is needed to validate these effects across diverse formulations and populations.(118)

9. Wound healing

Polyphenols aid wound healing by enhancing macrophage and fibroblast migration, improving microcirculation, and facilitating re-epithelialization through type I collagen deposition.(126,128,135)

**DIET PATTERNS / TYPES**

**1. WESTERN DIET**

Western diet has been implicated in the disruption of skin barrier function:

1. Role in oxidative stress

The Western diet is contributing to systemic oxidative stress.(136) Diets high in AGE-rich foods and SFAs promote oxidative stress, and elevated ROS levels contribute to the disruption of the skin barrier.(12,136,137) While the diet’s low intake of fruits and vegetables and extensive food processing, significantly diminishes the availability and efficacy of dietary antioxidants, impairs the skin’s capacity to mitigate oxidative damage.(138)

2. Affect protein function in the dermis

Diets high in AGE-rich foods promoting cross-linking of collagen fibers and affect protein function in the dermis.(12,137)

3. Lipid composition of the skin

A high-fat diet modifies the overall lipid composition of the skin.(139)

4. Skin microbiota

A high-fat diet is linked to alterations in skin microbiota.(139)

5. Role in immune regulation

The Western diet contributes to chronic inflammation and immune response dysregulation.(136) Specifically, an imbalance in the omega-6 to omega-3 fatty acid ratio favors pro-inflammatory pathways, including Th2-driven immune responses.(91) Furthermore, AGEs can exacerbate inflammatory processes by modulating IL-33 and TSLP levels.(140) Diets rich in AGE-containing foods further intensify chronic inflammation.(12,137) Frequent and excessive sugar consumption may result in chronically elevated blood glucose levels, which are associated with increased inflammatory responses.(141)

6. Role in gut-skin axis

The consumption of ultra-processed foods, often containing food additives and emulsifiers, has been associated with significant alterations in the gut microbiome, compromise of the gut epithelial barrier.(142) In mouse models, Western-type diets and high fructose intake have been shown to impair goblet cell function and reduce the mucus layer, thereby increasing gut permeability and altering microbial composition.(143,144) Added sugar and sugar-sweetened beverages also affect the gut microbiota,(145) while high-fat diets have been shown to decrease the expression of TJ, further exacerbating gut barrier dysfunction.(87,121,146) Diets high in fat also contribute to reduced microbial diversity and promote the accumulation of endotoxins, such as lipopolysaccharides, which can disrupt epithelial integrity, thin the mucus layer, and trigger the release of inflammatory mediators.(114) These factors collectively dysregulate immune system function and trigger systemic inflammation, adversely impacting skin health.(109) Additionally, the low pH and texture of commercial baby foods, particularly those with added fruit or citric acid, may impair epithelial integrity, especially with frequent consumption.(147) Collectively, these dietary factors contribute to immune dysregulation and systemic inflammation, which may adversely impact skin health (103).

7. Wound healing

A high-fat diet may impair the skin's ability to heal.(114)

**2. PLANT-BASED DIET**

1. Role in gut-skin axis

Research suggests that vegetarian and vegan diets promote a diverse and balanced gut microbiota, supporting both intestinal health.(148)

2. Wound healing

Vegan diet may present challenges to skin health, particularly in the context of wound healing. Proper cutaneous healing relies on adequate levels of proteins, amino acids, iron, and other essential nutrients at every stage of the repair process. Studies have noted that vegans may experience delayed wound healing, possibly due to insufficient intake of these key nutrients.(149)

**3. INTERMITTENT FASTING**

Intermittent fasting, an eating pattern with cycles of voluntary fasting and feeding, has been popular recently for losing weight, preventing cardiovascular and cerebrovascular diseases, and decresing the risk of diabetes and metabolic syndrome.

1. Role in immune regulation

Intermittent fasting has a boosting effect on immune memory together with anti-inflammatory effects.(150)

2. Role in gut-skin axis

Intermittent fasting also improves the diversity of gut microbiota by increasing the species of *Lactobacillus*, *Prevotella*, and *Bacteroidaceae*.(151) Recently it has also been shown that especially long term intermittent fasting (24h fasting/ 24h feeding) attenuates food allergy by reducing IL-4, IL-5, and IL-10 expressions, maintains intestinal barrier integrity by increasing ZO-1 expression, and also prevent gut dysbiosis in an OVA-induced food allergy mice model.(152) Allergens, contamination of microorganisms (bacteria, fungi, and viruses), emulsifiers in processed foods, and detergents may disrupt epithelial barrier and derives a Th2-mediated immune response.(142) Intermittent fasting has also the advantage of reducing contamination by these epithelial barrier disruptors.

**4. WATER CONSUMPTION**

Proper hydration through dietary water intake is essential for maintaining skin hydration and its biomechanical properties. Research indicates that increasing water intake as part of a regular diet may benefit normal skin physiology, especially in individuals with lower daily water consumption.(153)

REFERENCES

1. Trompette A, Ubags ND. Skin barrier immunology from early life to adulthood. Mucosal Immunol 2023;16:194-207.

2. Woodby B, Penta K, Pecorelli A et al. Skin Health from the Inside out. Annu Rev Food Sci Technol 2020;11:235-254.

3. Leung DYM, Berdyshev E, Goleva E. Cutaneous barrier dysfunction in allergic diseases. J Allergy and Clin Immunol 2020;145:1485-1497.

4. Yoshida T, Beck LA, De Benedetto A. Skin barrier defects in atopic dermatitis: From old idea to new opportunity. Allergol Int 2022;71:3-13.

5. Moreno-Macias H, Romieu I. Effects of antioxidant supplements and nutrients on patients with asthma and allergies. J Allergy and Clin Immunol 2014;133:1237-1244.

6. Maggini S, Beveridge S, Sorbara PJP et al. Feeding the immune system: The role of micronutrients in restoring resistance to infections. CAB Rev 2008;3.

7. Nosewicz J, Spaccarelli N, Roberts KM et al. The epidemiology, impact, and diagnosis of micronutrient nutritional dermatoses part 1: Zinc, selenium, copper, vitamin A, and vitamin C. J Am Acad Dermatol 2022;86:267-278.

8. Gombart AF, Pierre A, Maggini S. A Review of Micronutrients and the Immune System-Working in Harmony to Reduce the Risk of Infection. Nutrients 2020; 12(1):236

9. Pullar JM, Carr AC, Vissers MCM. The Roles of Vitamin C in Skin Health. Nutrients 2017;9:866.

10. Tourkochristou E, Triantos C, Mouzaki A. The Influence of Nutritional Factors on Immunological Outcomes. Front Immunol 2021;12:665968.

11. Munteanu C, Schwartz B. The relationship between nutrition and the immune system. Front Nutr 2022;9:1082500.

12. Muzumdar S, Ferenczi K. Nutrition and youthful skin. Clin Dermatol 2021;39:796-808.

13. Michalak M, Pierzak M, Kręcisz B et al. Bioactive Compounds for Skin Health: A Review. Nutrients 2021;13(1):203.

14. Ghani SMA, Goon JA, Azman NHEN et al. Comparing the effects of vitamin E tocotrienol-rich fraction supplementation and α-tocopherol supplementation on gene expression in healthy older adults. Clinics (Sao Paulo) 2019;74:e688

15. Wu D, Lewis ED, Pae M et al. Nutritional modulation of immune function: Analysis of evidence, mechanisms, and clinical relevance. Front Immunol 2019;10:431237.

16. Gao Y, Zhao C, Wang W et al. Prostaglandins E2 signal mediated by receptor subtype EP2 promotes IgE production in vivo and contributes to asthma development. Sci Rep. 2016;6:20505.

17. Banche G, Bracco P, Allizond V et al. Do Crosslinking and Vitamin E Stabilization Influence Microbial Adhesions on UHMWPE-based Biomaterials? Clin Orthop Relat Res 2015;473:974-986.

18. Cau L, Williams MR, Butcher AM et al. Staphylococcus epidermidis protease EcpA can be a deleterious component of the skin microbiome in atopic dermatitis. J Allergy Clin Immunol 2020;147:955.

19. Schagen SK, Zampeli VA, Makrantonaki E et al. Discovering the link between nutrition and skin aging. Dermatoendocrinol 2012;4:298-307.

20. Boelsma E, Van de Vijver LPL, Goldbohm RA et al. Human skin condition and its associations with nutrient concentrations in serum and diet. Am J Clin Nutr 2003;77:348-355.

21. Zasada M, Budzisz E. Retinoids: active molecules influencing skin structure formation in cosmetic and dermatological treatments. Postepy Dermatol Alergol. 2019;36(4):392-397.

22. Telgenhoff D, Ramsay S, Hilz S et al. Claudin 2 mRNA and Protein Are Present in Human Keratinocytes and May Be Regulated by All-trans- Retinoic Acid. Skin Pharmacol Physiol 2008;21:211-217.

23. Yokota-Nakatsuma A, Takeuchi H, Ohoka Y et al. Retinoic acid prevents mesenteric lymph node dendritic cells from inducing IL-13-producing inflammatory Th2 cells. Mucosal Immunol 2014;7:786-801.

24. Zhao J, Lloyd CM, Noble A. Th17 responses in chronic allergic airway inflammation abrogate regulatory T-cell-mediated tolerance and contribute to airway remodeling. Mucosal Immunol 2013;6:335-346.

25. Cox SE, Arthur P, Kirkwood BR et al. Vitamin A supplementation increases ratios of proinflammatory to anti-inflammatory cytokine responses in pregnancy and lactation. Clin Exp Immunol 2006;144:392-400.

26. Scheffel F, Heine G, Henz BM et al. Retinoic acid inhibits CD40 plus IL-4 mediated IgE production through alterations of sCD23, sCD54 and IL-6 production. Inflamm Res 2005;54:113-118.

27. Julia V, Macia L, Dombrowicz D. The impact of diet on asthma and allergic diseases. Nat Rev Immunol 2015;15:308-322.

28. Vassilopoulou E, Venter C, Roth-Walter F. Malnutrition and Allergies: Tipping the Immune Balance towards Health. J Clin Med 2024;13(16):4713.

29. He C, Deng J, Hu X et al. Vitamin A inhibits the action of LPS on the intestinal epithelial barrier function and tight junction proteins. Food Funct 2019;10:1235-1242.

30. Peterson CT, Rodionov DA, Peterson SN et al. B Vitamins and Their Role in Immune Regulation and Cancer. Nutrients 2020;12(11):3380.

31. Nosewicz J, Spaccarelli N, Roberts KM et al. The epidemiology, impact, and diagnosis of micronutrient nutritional dermatoses. Part 2: B-complex vitamins. J Am Acad Dermatol 2022;86:281-292.

32. Marques C, Hadjab F, Porcello A et al. Mechanistic Insights into the Multiple Functions of Niacinamide: Therapeutic Implications and Cosmeceutical Applications in Functional Skincare Products. Antioxidants (Basel) 2024;13(4):425.

33. McCusker MM, Grant-Kels JM. Healing fats of the skin: the structural and immunologic roles of the omega-6 and omega-3 fatty acids. Clin Dermatol 2010;28:440-451.

34. Tricarico PM, Mentino D, De Marco A et al. Aquaporins Are One of the Critical Factors in the Disruption of the Skin Barrier in Inflammatory Skin Diseases. Int J Mol Sci 2022;23(7):4020.

35. Makino E, Fukuyama T, Watanabe Y et al. Subacute oral administration of folic acid elicits anti-inflammatory response in a mouse model of allergic dermatitis. J Nutr Biochem 2019;67:14-19.

36. Kamanna VS, Ganji SH, Kashyap ML. The mechanism and mitigation of niacin-induced flushing. Int J Clin Pract 2009;63:1369-1377.

37. Tomova A, Bukovsky I, Rembert E et al. The Effects of Vegetarian and Vegan Diets on Gut Microbiota. Front Nutr 2019;6:47.

38. Bedani R, Cucick ACC, Albuquerque MAC de et al. B-Group Vitamins as Potential Prebiotic Candidates: Their Effects on the Human Gut Microbiome. J Nutr 2024;154:341-353.

39. Grant ET, Parrish A, Boudaud M et al. Dietary fibers boost gut microbiota-produced B vitamin pool and alter host immune landscape. Microbiome 2024;12(1):179.

40. Chen AC, Martin AJ, Dalziell RA et al. Oral nicotinamide reduces transepidermal water loss: a randomized controlled trial. Br J Dermatol 2016;175:1363-1365.

41. Gordon-Thomson C, Tongkao-On W, Song EJ et al. Protection from Ultraviolet Damage and Photocarcinogenesis by Vitamin D Compounds. Adv Exp Med Biol 2014;810:303-328.

42. Bikle DD, Chang S, Crumrine D et al. 25 Hydroxyvitamin D 1 α-hydroxylase is required for optimal epidermal differentiation and permeability barrier homeostasis. J Invest Dermatol. 2004;122(4):984-992.

43. Gniadecki R, Gajkowska B, Hansen M. 1,25-Dihydroxyvitamin D3 Stimulates the Assembly of Adherens Junctions in Keratinocytes: Involvement of Protein Kinase C. Endocrinology 1997;138:2241-2248.

44. Chen L, Dong Y, Bhagatwala J et al. Vitamin D3 Supplementation Increases Long-Chain Ceramide Levels in Overweight/Obese African Americans: A Post-Hoc Analysis of a Randomized Controlled Trial. Nutrients 2020;12(4):981.

45. Oda Y, Uchida Y, Moradian S et al. Vitamin D receptor and coactivators SRC2 and 3 regulate epidermis-specific sphingolipid production and permeability barrier formation. J Invest Dermatol 2009;129(6):1367-1378.

46. Sadeghi K, Wessner B, Laggner U et al. Vitamin D3 down-regulates monocyte TLR expression and triggers hyporesponsiveness to pathogen-associated molecular patterns. Eur J Immunol 2006;36:361-370.

47. Karin M, Lin A. NF-kappaB at the crossroads of life and death. Nat Immunol 2002;3:221-227.

48. Hartmann B, Heine G, Babina M et al. Targeting the vitamin D receptor inhibits the B cell-dependent allergic immune response. Allergy 2011;66:540-548.

49. Biedrzycki G, Wolszczak-Biedrzycka B, Dorf J et al. The antioxidant barrier, oxidative/nitrosative stress, and protein glycation in allergy: from basic research to clinical practice. Front Immunol 2024;15:1440313.

50. Segaert S. Vitamin D regulation of cathelicidin in the skin: toward a renaissance of vitamin D in dermatology? J Invest Dermatol 2008;128:773-775.

51. Razzaghi R, Pourbagheri H, Momen-Heravi M et al. The effects of vitamin D supplementation on wound healing and metabolic status in patients with diabetic foot ulcer: A randomized, double-blind, placebo-controlled trial. J Diabetes Complications 2017;31:766-772.

52. Wright JA, Richards T, Srai SKS. The role of iron in the skin and cutaneous wound healing. Front Pharmacol 2014;5:156.

53. Roth-Walter F. Iron-Deficiency in Atopic Diseases: Innate Immune Priming by Allergens and Siderophores. Front Allergy 2022;3:859922.

54. Peroni DG, Hufnagl K, Comberiati P et al. Lack of iron, zinc, and vitamins as a contributor to the etiology of atopic diseases. Front Nutr 2023;9:1032481.

55. Shaheen SO, Macdonald-Wallis C, Lawlor DA et al. Haemoglobin concentrations in pregnancy and respiratory and allergic outcomes in childhood: Birth cohort study. Clin Exp Allergy 2017;47(12):1615-1624.

56. Le HT, Brouwer ID, Nguyen KC et al. The effect of iron fortification and de-worming on anaemia and iron status of Vietnamese schoolchildren. Br J Nutr 2007;97(5):955-962.

57. Protudjer JLP, Roth-Walter F, Meyer R. Nutritional Considerations of Plant-Based Diets for People With Food Allergy. Clin Exp Allergy 2024;54(11):895-908.

58. Podgórska A, Kicman A, Naliwajko S et al. Zinc, Copper, and Iron in Selected Skin Diseases. Int J Mol Sci 2024;25(7):3823.

59. Ogawa Y, Kawamura T, Shimada S. Zinc and skin biology. Arch Biochem Biophys 2016;611:113-119.

60. Ogawa Y, Kinoshita M, Shimada S et al. Zinc in Keratinocytes and Langerhans Cells: Relevance to the Epidermal Homeostasis. J Immunol Res 2018;2018:5404093.

61. Choi EH, Kang H. Importance of Stratum Corneum Acidification to Restore Skin Barrier Function in Eczematous Diseases. Ann Dermatol 2024;36:1-8.

62. Liu MJ, Bao S, Gálvez-Peralta M et al. ZIP8 regulates host defense through zinc-mediated inhibition of NF-κB. Cell Rep 2013;3:386-400.

63. Brieger A, Rink L, Haase H. Differential regulation of TLR-dependent MyD88 and TRIF signaling pathways by free zinc ions. J Immunol 2013;191:1808-1817.

64. Wessels I, Maywald M, Rink L. Zinc as a Gatekeeper of Immune Function. Nutrients 2017;9(12):1286.

65. Gao H, Dai W, Zhao L et al. The Role of Zinc and Zinc Homeostasis in Macrophage Function. J Immunol Res 2018;2018:6872621.

66. Finamore A, Massimi M, Devirgiliis LC et al. Zinc deficiency induces membrane barrier damage and increases neutrophil transmigration in Caco-2 cells. J Nutr 2008;138:1664-1670.

67. Lin PH, Sermersheim M, Li H et al. Zinc in Wound Healing Modulation. Nutrients 2017;10(1):16.

68. Altobelli GG, Van Noorden S, Balato A et al. Copper/Zinc Superoxide Dismutase in Human Skin: Current Knowledge. Front Med (Lausanne) 2020;7:537401.

69. Borkow G. Using Copper to Improve the Well-Being of the Skin. Curr Chem Biol 2015;8:89-102.

70. Focarelli F, Giachino A, Waldron KJ. Copper microenvironments in the human body define patterns of copper adaptation in pathogenic bacteria. PLoS Pathog 2022;18:e1010617.

71. Jobeili L, Rousselle P, Béal D et al. Selenium preserves keratinocyte stemness and delays senescence by maintaining epidermal adhesion. Aging 2017;9:2302-2315.

72. Zhang Y, Roh YJ, Han SJ et al. Role of Selenoproteins in Redox Regulation of Signaling and the Antioxidant System: A Review. Antioxidants (Basel) 2020;9(5):383.

73. Zhu X, Jiang M, Song E et al. Selenium deficiency sensitizes the skin for UVB-induced oxidative damage and inflammation which involved the activation of p38 MAPK signaling. Food Chem Toxicol 2015;75:139-145.

74. Sengupta A, Lichti UF, Carlson BA et al. Selenoproteins Are Essential for Proper Keratinocyte Function and Skin Development. PLoS One 2010;5:e12249.

75. Heidar Z, Hamzepour N, Zadeh Modarres S et al. The Effects of Selenium Supplementation on Clinical Symptoms and Gene Expression Related to Inflammation and Vascular Endothelial Growth Factor in Infertile Women Candidate for In Vitro Fertilization. Biol Trace Elem Res 2020;193:319-325.

76. Yamane T, Konno R, Iwatsuki K et al. Protein-restricted maternal diet during lactation decreases type I and type III tropocollagen synthesis in the skin of mice offspring. Biosci Biotechnol Biochem 2018;82:1829-1831.

77. Li P, Yin YL, Li D et al. Amino acids and immune function. Br J Nutr 2007;98:237-252.

78. Markova M, Koelman L, Hornemann S et al. Effects of plant and animal high protein diets on immune-inflammatory biomarkers: A 6-week intervention trial. Clin Nutr 2020;39:862-869.

79. Rubio-Patiño C, Bossowski JP, De Donatis GM et al. Low-Protein Diet Induces IRE1α-Dependent Anticancer Immunosurveillance. Cell Metab 2018;27:828-842.e7.

80. Tang Y, Li J, Liao S et al. The effect of dietary protein intake on immune status in pigs of different genotypes. Food Agric Immunol 2018;29:776-784.

81. Qin Z, Wang Y, Zhao W et al. Pressure ulcer healing promoted by adequate protein intake in rats. Exp Ther Med 2018;15:4173.

82. Niemiro GM, Chiarlitti NA, Khan NA et al. A Carbohydrate Beverage Reduces Monocytes Expressing TLR4 in Children with Overweight or Obesity. J Nutr 2020;150:616-622.

83. Nieman DC. Nutrition, exercise, and immune system function. Clin Sports Med 1999;18:537-548.

84. Zhou S, Zhu W, Qin X et al. Synthesis and Evaluation of Antioxidant and Potential Prebiotic Activities of Acetylated and Butyrylated Fructo-Oligosaccharides. Antioxidants (Basel) 2022;11(9):1658.

85. Yue X, Wen S, Long-kun D et al. Three important short-chain fatty acids (SCFAs) attenuate the inflammatory response induced by 5-FU and maintain the integrity of intestinal mucosal tight junction. BMC Immunol 2022;23:1-13.

86. Kapoor MP, Yamaguchi H, Ishida H et al. The effects of prebiotic partially hydrolyzed guar gum on skin hydration: A randomized, open-label, parallel, controlled study in healthy humans. J Funct Foods 2023;103:105494.

87. Salem I, Ramser A, Isham N et al. The gut microbiome as a major regulator of the gut-skin axis. Front Microbiol 2018;9:382698.

88. Djuricic I, Calder PC. Beneficial Outcomes of Omega-6 and Omega-3 Polyunsaturated Fatty Acids on Human Health: An Update for 2021. Nutrients 2021;13(7):2421.

89. Jia T, Qiao W, Yao Q et al. Treatment with Docosahexaenoic Acid Improves Epidermal Keratinocyte Differentiation and Ameliorates Inflammation in Human Keratinocytes and Reconstructed Human Epidermis Models. Molecules 2019;24(17):3156.

90. Fujii M, Ohyanagi C, Kawaguchi N et al. Eicosapentaenoic acid ethyl ester ameliorates atopic dermatitis-like symptoms in special diet-fed hairless mice, partly by restoring covalently bound ceramides in the stratum corneum. Exp Dermatol 2018;27:837-840.

91. Celebi Sozener Z, Özbey Yücel Ü, Altiner S et al. The External Exposome and Allergies: From the Perspective of the Epithelial Barrier Hypothesis. Frontiers in Allergy 2022;3:887672.

92. Pilkington SM, Watson REB, Nicolaou A et al. Omega-3 polyunsaturated fatty acids: photoprotective macronutrients. Exp Dermatol 2011;20:537-543.

93. Li K, Huang T, Zheng J et al. Effect of marine-derived n-3 polyunsaturated fatty acids on C-reactive protein, interleukin 6 and tumor necrosis factor α: a meta-analysis. PLoS One 2014;9(2):e88103.

94. Sui YH, Luo WJ, Xu QY et al. Dietary saturated fatty acid and polyunsaturated fatty acid oppositely affect hepatic NOD-like receptor protein 3 inflammasome through regulating nuclear factor-kappa B activation. World J Gastroenterol 2016;22:2533-2544.

95. MacLean E, Madsen N, Vliagoftis H et al. n-3 Fatty acids inhibit transcription of human IL-13: implications for development of T helper type 2 immune responses. Br J Nutr 2013;109:990-1000.

96. D’Vaz N, Meldrum SJ, Dunstan JA et al. Fish oil supplementation in early infancy modulates developing infant immune responses. Clin Exp Allergy 2012;42:1206-1216.

97. Han SC, Kang GJ, Ko YJ et al. Fermented fish oil suppresses T helper 1/2 cell response in a mouse model of atopic dermatitis via generation of CD4+CD25+Foxp3+ T cells. BMC Immunol 2012;13:44.

98. Van Den Elsen LWJ, Nusse Y, Balvers M et al. n-3 Long-chain PUFA reduce allergy-related mediator release by human mast cells in vitro via inhibition of reactive oxygen species. Br J Nutr 2013;109:1821-1831.

99. De Spirt S, Stahl W, Tronnier H et al. Intervention with flaxseed and borage oil supplements modulates skin condition in women. Br J Nutr 2009;101:440-445.

100. Seethaler B, Lehnert K, Yahiaoui-Doktor M et al. Omega-3 polyunsaturated fatty acids improve intestinal barrier integrity—albeit to a lesser degree than short-chain fatty acids: an exploratory analysis of the randomized controlled LIBRE trial. Eur J Nutr 2023;62:2779-2791.

101. Barcelos RCS, de Mello-Sampayo C, Antoniazzi CTD et al. Oral supplementation with fish oil reduces dryness and pruritus in the acetone-induced dry skin rat model. J Dermatol Sci 2015;79:298-304.

102. Neukam K, De Spirt S, Stahl W et al. Supplementation of flaxseed oil diminishes skin sensitivity and improves skin barrier function and condition. Skin Pharmacol Physiol 2011;24:67-74.

103. Silva JR, Burger B, Kühl CMC et al. Wound Healing and Omega-6 Fatty Acids: From Inflammation to Repair. Mediators Inflamm 2018;2018:2503950.

104. Romana-Souza B, Monte-Alto-Costa A. Olive oil reduces chronic psychological stress-induced skin aging in mice through the NF-κB and NRF2 pathways. J Funct Foods 2019;54:310-319.

105. Donato-Trancoso A, Monte-Alto-Costa A, Romana-Souza B. Olive oil-induced reduction of oxidative damage and inflammation promotes wound healing of pressure ulcers in mice. J Dermatol Sci 2016;83:60-69.

106. Najmi M, Shariatpanahi ZV, Tolouei M et al. Effect of oral olive oil on healing of 10-20% total body surface area burn wounds in hospitalized patients. Burns 2015;41:493-496.

107. González F, Considine R V., Abdelhadi OA et al. Oxidative Stress in Response to Saturated Fat Ingestion Is Linked to Insulin Resistance and Hyperandrogenism in Polycystic Ovary Syndrome. J Clin Endocrinol Metab 2019;104:5360-5371.

108. Rocha DM, Caldas AP, Oliveira LL et al. Saturated fatty acids trigger TLR4-mediated inflammatory response. Atherosclerosis 2016;244:211-215.

109. Fritsche KL. The science of fatty acids and inflammation. Adv Nutr 2015;6:293S-301S.

110. Li J, Wang Y, Tang L et al. Dietary medium-chain triglycerides promote oral allergic sensitization and orally induced anaphylaxis to peanut protein in mice. J Allergy Clin Immunol 2013;131:442-450.

111. Barcelos RCS, Vey LT, Segat HJ et al. Influence of trans fat on skin damage in first-generation rats exposed to UV radiation. Photochem Photobiol 2015;91:424-430.

112. Ghanim H, Abuaysheh S, Sia CL et al. Increase in Plasma Endotoxin Concentrations and the Expression of Toll-Like Receptors and Suppressor of Cytokine Signaling-3 in Mononuclear Cells After a High-Fat, High-Carbohydrate MealImplications for insulin resistance. Diabetes Care 2009;32:2281-2287.

113. Lopez-Garcia E, Schulze MB, Meigs JB et al. Consumption of Trans Fatty Acids Is Related to Plasma Biomarkers of Inflammation and Endothelial Dysfunction. J Nutr 2005;135:562-566.

114. Mahmud MR, Akter S, Tamanna SK et al. Impact of gut microbiome on skin health: gut-skin axis observed through the lenses of therapeutics and skin diseases. Gut Microbes 2022;14(1):2096995.

115. Mori N, Kano M, Masuoka N et al. Effect of probiotic and prebiotic fermented milk on skin and intestinal conditions in healthy young female students. Biosci Microbiota Food Health 2016;35(3):105-112.

116. Kano M, Masuoka N, Kaga C, et al. Consecutive Intake of Fermented Milk Containing Bifidobacterium breve Strain Yakult and Galacto-oligosaccharides Benefits Skin Condition in Healthy Adult Women. Biosci Microbiota Food Health 2013;32(1):33-39

117. Strouphauer E, Parke M, Perez-Sanchez A et al. Functional Foods in Dermatology. Dermatol Pract Concept 2023;13:e2023256-e2023256.

118. Sun Q, Wu J, Qian G et al. Effectiveness of Dietary Supplement for Skin Moisturizing in Healthy Adults: A Systematic Review and Meta-Analysis of Randomized Controlled Trials. Front Nutr 2022;9:895192.

119. Taleb S. Tryptophan Dietary Impacts Gut Barrier and Metabolic Diseases. Front Immunol 2019;10:2113.

120. Hou Y, Li J, Ying S. Tryptophan Metabolism and Gut Microbiota: A Novel Regulatory Axis Integrating the Microbiome, Immunity, and Cancer. Metabolites 2023;13:1166.

121. Roduit C, Frei R, Ferstl R et al. High levels of butyrate and propionate in early life are associated with protection against atopy. Allergy 2019;74:799-809.

122. Losol P, Wolska M, Wypych TP et al. A cross talk between microbial metabolites and host immunity: Its relevance for allergic diseases. Clin Transl Allergy 2024;14:e12339.

123. Venter C, Meyer RW, Greenhawt M et al. Role of dietary fiber in promoting immune health-An EAACI position paper. Allergy 2022;77:3185-3198.

124. Chen Y, Li Y, Li X et al. Indole‑3‑propionic acid alleviates intestinal epithelial cell injury via regulation of the TLR4/NF‑κB pathway to improve intestinal barrier function. Mol Med Rep 2024;30(4):189.

125. Li J, Zhang L, Wu T et al. Indole-3-propionic Acid Improved the Intestinal Barrier by Enhancing Epithelial Barrier and Mucus Barrier. J Agric Food Chem 2021;69:1487-1495.

126. Sun M, Deng Y, Cao X et al. Effects of Natural Polyphenols on Skin and Hair Health: A Review. Molecules 2022;27(22):7832.

127. Sticozzi C, Belmonte G, Cervellati F et al. Resveratrol protects SR-B1 levels in keratinocytes exposed to cigarette smoke. Free Radic Biol Med 2014;69:50-57.

128. Wen S, Zhang J, Yang B et al. Role of Resveratrol in Regulating Cutaneous Functions. Evid Based Complement Alternat Med 2020;2020:2416837.

129. Di Salvo E, Gangemi S, Genovese C et al. Polyphenols from Mediterranean Plants: Biological Activities for Skin Photoprotection in Atopic Dermatitis, Psoriasis, and Chronic Urticaria. Plants (Basel) 2023;12(20):3579.

130. Zhang Z, Zhao Y, Han Y et al. The natural substances with anti-allergic properties in food allergy. Trends Food Sci Technol 2022;128:53-67.

131. Sozmen SC, Karaman M, Micili SC et al. Resveratrol ameliorates 2,4-dinitrofluorobenzene-induced atopic dermatitis-like lesions through effects on the epithelium. PeerJ 2016;2016:e1889.

132. Singh A, Yau YF, Leung KS et al. Interaction of Polyphenols as Antioxidant and Anti-Inflammatory Compounds in Brain-Liver-Gut Axis. Antioxidants 2020;9:669.

133. Yang G, Bibi S, Du M et al. Regulation of the intestinal tight junction by natural polyphenols: A mechanistic perspective. Crit Rev Food Sci Nutr 2017;57:3830-3839.

134. Plamada D, Vodnar DC. Polyphenols—Gut Microbiota Interrelationship: A Transition to a New Generation of Prebiotics. Nutrients 2021;14:137.

135. Kwon AH, Qiu Z, Hashimoto M et al. Effects of medicinal mushroom (Sparassis crispa) on wound healing in streptozotocin-induced diabetic rats. Am J Surg 2009;197:503-509.

136. Clemente-Suárez VJ, Beltrán-Velasco AI, Redondo-Flórez L et al. Global Impacts of Western Diet and Its Effects on Metabolism and Health: A Narrative Review. Nutrients 2023;15(12):2749.

137. Berni Canani R, Carucci L, Coppola S et al. Ultra-processed foods, allergy outcomes and underlying mechanisms in children: An EAACI task force report. Pediatr Allergy Immunol 2024;35(9):e14231.

138. Toydemir G, Gultekin Subasi B, Hall RD et al. Effect of food processing on antioxidants, their bioavailability and potential relevance to human health. Food Chem X 2022;14:100334.

139. Moestrup KS, Chen Y, Schepeler T et al. Dietary Control of Skin Lipid Composition and Microbiome. J Invest Dermatol 2018;138:1225-1228.

140. Uribarri J, Woodruff S, Goodman S et al. Advanced Glycation End Products in Foods and a Practical Guide to Their Reduction in the Diet. J Am Diet Assoc 2010;110:911-916.e12.

141. Ma X, Nan F, Liang H et al. Excessive intake of sugar: An accomplice of inflammation. Front Immunol 2022;13:988481.

142. Akdis CA. Does the epithelial barrier hypothesis explain the increase in allergy, autoimmunity and other chronic conditions? Nat Rev Immunol 2021;21:739-751.

143. Volynets V, Louis S, Pretz D et al. Intestinal Barrier Function and the Gut Microbiome Are Differentially Affected in Mice Fed a Western-Style Diet or Drinking Water Supplemented with Fructose. J Nutr 2017;147:770-780.

144. David LA, Maurice CF, Carmody RN et al. Diet rapidly and reproducibly alters the human gut microbiome. Nature 2013;505:559.

145. Ramne S, Brunkwall L, Ericson U et al. Gut microbiota composition in relation to intake of added sugar, sugar-sweetened beverages and artificially sweetened beverages in the Malmö Offspring Study. Eur J Nutr 2021;60:2087-2097.

146. Cremonini E, Wang Z, Bettaieb A et al. (-)-Epicatechin protects the intestinal barrier from high fat diet-induced permeabilization: Implications for steatosis and insulin resistance. Redox Biol 2018;14:588-599.

147. Knight T, Smith PK, Soutter V et al. Is the low pH of infant and toddler foods a concern? Pediatr Allergy Immunol 2021;32:1103-1106.

148. Tomova A, Bukovsky I, Rembert E et al. The effects of vegetarian and vegan diets on gut microbiota. Front Nutr 2019;6:447652.

149. Fusano M, Zane C, Calzavara-Pinton PG et al. Photodynamic therapy for actinic keratosis in vegan and omnivore patients: the role of diet on skin healing. J Dermatolog Treat 2021;32:78-83.

150. Okawa T, Nagai M, Hase K. Dietary Intervention Impacts Immune Cell Functions and Dynamics by Inducing Metabolic Rewiring. Front Immunol 2021;11:623989.

151. Cignarella F, Cantoni C, Ghezzi L et al. Intermittent Fasting Confers Protection in CNS Autoimmunity by Altering the Gut Microbiota. Cell Metab 2018;27:1222-1235.e6.

152. Ma RX, Hu JQ, Fu W et al. Intermittent fasting protects against food allergy in a murine model via regulating gut microbiota. Front Immunol 2023;14:1167562.

153. Palma L, Marques LT, Bujan J et al. Dietary water affects human skin hydration and biomechanics. Clin Cosmet Investig Dermatol 2015;8:413-421.
